# *OsWHY1* Negatively Modulates Rice Immunity Against *Magnaporthe oryzae*

**DOI:** 10.3389/fpls.2026.1931455

**Published:** 2026-09-28

**Authors:** Xuanning Zeng, Weiye Peng, Na Song, Yuqiang Zhao, Chenting Huang, Kunjin Zhou, Wei Li, Liangying Dai, Bing Wang

**Affiliations:** 1 College of Plant Protection, Hunan Agricultural University, Changsha, Hunan, China; 2 Hunan Plant Protection Institute, Hunan Academy of Agricultural Science, Changsha, Hunan, China

**Keywords:** *Oryza sativa*, *Magnaporthe oryzae*, OsWHY1, PTI, susceptibility, transcriptome

## Abstract

WHIRLY proteins, known for their DNA/RNA-binding capabilities conferred by a highly conserved WHIRLY domain, are integral to plant defense mechanisms. However, the role of OsWHY1 in rice immunity remains to be clarified. We examined OsWHY1 expression after treatment with exogenous hormones, PAMPs, and Magnaporthe oryzae. We generated OsWHY1-overexpressing rice plants and performed yeast two-hybrid (Y2H), bimolecular fluorescence complementation (BiFC), transcriptome, and RT-qPCR analyses. OsWHY1 expression was upregulated by exogenous hormones and PAMPs, with a significant increase at 24 h post-inoculation with M. oryzae. Overexpression of OsWHY1 correlated with reduced resistance to M. oryzae. Y2H and BiFC assays demonstrated that OsWHY1 could form homomeric complexes within the chloroplast, and OsWHY1 did not possess transcriptional activation activity. Transcriptome profiling of OsWHY1-overexpressing plants revealed significant downregulation of genes associated with glutathione transferase activity. Chitin-induced reactive oxygen species (ROS) accumulation was diminished in OsWHY1-overexpressing plants compared with the Nipponbare (NPB) control. The transcriptional levels of key stress- and defense-related genes, including OsWAK1, OsWRKY53, OsPR2, OsSAG12, OsHSP58.7, OsCATB and OsMYB1R, were markedly downregulated in OsWHY1-overexpressing plants. Collectively, these findings indicate that OsWHY1 functions as a negative regulator of rice immunity against M. oryzae by suppressing the expression of defense-related genes. Thus, our study identifies OsWHY1 as a negative immune regulator and suggests that its manipulation could be a strategy for breeding blast-resistant rice varieties.

## 1 Introduction

In order to counteract potential pathogens, plants have developed intricate defense mechanisms, such as molecules to initiate PAMP-triggered immunity (PTI). PTI is triggered by the recognition of pathogen-derived molecules via cell surface-localized pattern-recognition receptors (Yuan et al., 2021). This process is followed by a complex orchestration involving the biosynthesis, transport, and metabolism of multiple hormones, as well as the interplay of their signaling pathways, transcription factors, and responsive genes, all of which contribute to the plant’s defense mechanisms (Simura et al., 2018). PTI results in a cascade of immune responses, including the rapid production of reactive oxygen species (ROS), the activation of mitogen-activated protein kinases (MAPKs), and the induction of pathogenesis-related (*PR*) gene expression (Liu et al., 2021).

Plant chloroplasts are essential organelles within plant cells, participating in a multitude of biological processes, including defense mechanisms. They serve as a significant source of defense signaling molecules, such as reactive oxygen species (ROS), reactive nitrogen oxide intermediates (NOIs), salicylic acid (SA), and jasmonic acid (JA). Consequently, pathogens may secrete certain effector proteins into the chloroplasts to dampen the plant’s immune response (Zhao et al., 2021). Moreover, the chloroplast outer envelope protein constitutively induces stormless in the absence of pathogen infection and enhances programmed cell death (Caplan et al., 2015). Recent studies have highlighted that WHIRLY proteins play a key role in the organization and function of chloroplasts (Prikryl et al., 2008; Krupinska et al., 2014; Oetke et al., 2022; Qiu et al., 2022).

WHIRLY proteins are a class of DNA/RNA-binding proteins characterized by a highly conserved WHIRLY DNA-binding domain, which is present in all DNA-containing compartments of plant cells. In higher plants, a common KGKAAL motif within the WHIRLY domain has been identified, which is instrumental for binding to single-stranded DNA (ssDNA). Notably, the presence of the conserved WHIRLY domain is both a necessary and sufficient condition for effective ssDNA binding (Huang et al., 2022; Krupinska et al., 2022). The KGKAAL motif-mediated ssDNA binding implies that WHIRLY proteins may directly regulate gene expression by binding to promoter regions of defense-related genes. The first WHIRLY protein to be characterized was the *PR-10a* binding factor 2 (PBF-2), which interacts with the promoter of the potato *PR-10a* gene. Crystallographic studies have shown that PBF-2 adopts a tetrameric structure reminiscent of a vortex, with each subunit weighing approximately 24 kDa (Desveaux et al., 2000). Comparative structural analysis of WHIRLY proteins from both potato and *Arabidopsis thaliana* has revealed a conserved pattern of specific residues that form a binding platform for ssDNA in a non-sequence-specific manner (Desveaux et al., 2005). WHIRLY proteins interact extensively with organelle proteins and nuclear transcription factors, and they play a role in organelle-nucleus communication. Therefore, they are posited to coordinate various cellular processes and contribute significantly to plant development (Karpinska et al., 2022). In recent years, WHIRLY proteins have attracted considerable interest for their roles in abiotic stress tolerance. They have been implicated in responses to hydrogen peroxide (H_2_O_2_), cold, and drought (Lin et al., 2019; Zhuang et al., 2019; Yan et al., 2020; Ruan et al., 2022).

WHIRLY proteins across various plant species have demonstrated a significant role in conferring resistance to biotic stresses and countering pathogen attacks. WHIRLY proteins show species- and context-dependent roles in immunity, ranging from positive to negative regulation. For instance, the StWhy1 protein in potato can stimulate the expression of the *PR-10a* gene during infection by the late blight oomycete pathogen, Phytophthora infestans, or in response to the elicitor arachidonic acid (Despres et al., 1995). In *Arabidopsis thaliana*, AtWHY1 is recognized as a key component of the salicylic acid (SA)-mediated, but NPR1-independent, pathway that governs disease resistance (Desveaux et al., 2004). The MeWHYs proteins in cassava have been shown to enhance disease resistance against bacterial blight by forming an interactive complex with MeWRKY75, specifically MeWHY1/2/3 (Liu et al., 2018). Additionally, VvWhy1 in grapevine has been identified to bolster resistance to *Phytophthora capsica* (Lai et al., 2022). Conversely, NbWhy1 in *Nicotiana benthamiana* has been found to negatively modulate the plant’s response to infection by Tomato yellow leaf curl China virus (TYLCCNV) and Tomato yellow leaf curl Brazil virus (TYLCCNB) (Sun et al., 2023).

Rice (*Oryza sativa* L.) stands as one of the world’s most crucial crops, providing sustenance for an estimated 4 billion individuals. However, rice blast disease, caused by the fungus *Magnaporthe oryzae*, poses a substantial threat to global rice yields (Oses-Ruiz et al., 2021; Chen et al., 2022). Nevertheless, the function of WHIRLY proteins in rice and their specific role in *M. oryzae* interaction remained unknown. In this study, we identified that OsWHY1 plays a detrimental role in the rice plant’s immune response to *M. oryzae* infection. Subsequent biochemical and transcriptomic analyses revealed that OsWHY1 negatively regulates defense-related gene expression and signaling pathways, resulting in enhanced susceptibility to the pathogen.

## 2 Materials and methods

### 2.1 Plant materials and growth conditions

The rice materials used in this study included transgenic plants overexpressing the *OsWHY1* gene, all in the genetic background of the cultivar ‘Nipponbare’ (NPB, *Oryza sativa* ssp. *japonica*). Overexpression lines were generated by cloning the full-length coding sequence (CDS) driven by the maize *Ubiquitin* promoter into the pCXUN-HA vector. Additionally, CRISPR/Cas9-mediated *oswhy1* heterozygous mutants (*OsWHY1/oswhy1*) were derived from the japonica rice cultivar ‘Zhonghua 11’. Transgenic plants from the T2 generation and subsequent progeny were selected on half-strength Murashige and Skoog (1/2 MS) medium containing 50 mg/L hygromycin for 5 days. Following germination, the rice seedlings were transplanted into pots with sterilized nutrient soil and grown in a greenhouse under conditions of 28 °C with a 16-hour light/8-hour dark photoperiod.

### 2.2 RNA extraction, cDNA synthesis, Primer design and quantitative real-time PCR

Total RNA was isolated using the PureLink RNA Mini Kit (Invitrogen, Beijing, China) following the manufacturer’s protocol (Peng et al., 2021b). First-strand cDNA was synthesized using a reverse transcription system (Waltham, MA, USA) according to the manufacturer’s instructions. RNA quality and quantity were assessed by agarose gel electrophoresis and spectrophotometry. Primers for the target genes were designed using the Primer-BLAST tool, available at the National Center for Biotechnology Information (http://www.ncbi.nlm.nih.gov/tools/primer-blast/). All primer sequences are listed in **Supplementary Table S1**. Quantitative real-time polymerase chain reaction (RT-qPCR) was performed as previously described (Li et al., 2023). The mRNA level of each gene was normalized to the transcript level of the reference gene *Oryza sativa Ubiquitin* (*OsUBQ*). Relative expression was calculated using the RT-qPCR delta-delta CT method, as per established protocols. Statistical analysis was performed using Student’s t-test, with significance set at * *P* < 0.05 and ** *P* < 0.01.

### 2.3 Rice blast inoculation assays

Rice blast inoculation was performed as previously described (Peng et al., 2023). *M. oryzae* isolate 110-2, preserved in our laboratory was cultured at 28 °C on tomato-oat agar medium. Cultures were initially incubated in darkness for one week, followed by a photoperiod to induce conidial formation for 5 to 7 days. The spore concentration was determined using a hemocytometer under a light microscope, and the suspension was adjusted to a final concentration of approximately 5 × 10^5^ spores/mL with 0.1% (v/v) Tween-20 solution. For punch inoculation, fully expanded leaves from seven-week-old rice seedlings were punctured with a sterile punch tool, and 10 μL of the conidia suspension were applied to the wounded sites. The inoculation sites were then covered with transparent surgical tape to maintain humidity. Three-leaf stage rice seedlings were subjected to spray inoculation. Following inoculation, the rice plants were placed in a dark, humidified incubator for 24 hours before being transferred to a solar greenhouse for disease progression over one week (for spray-inoculated plants) or two weeks (for punch-inoculated plants). Lesion length on detached leaves or lesion number on the inoculated plants was assessed at 5-day post-inoculation (dpi). In planta fungal growth was quantified by RT-qPCR measuring the relative abundance of the *M. oryzae Pot2* gene (*MoPot2*) normalized to the *OsUBQ* reference gene. Each treatment consisted of three biological replicates.

### 2.4 Flg22 and chitin treatment

At the three-leaf, one-tiller stage, uniformly grown wild-type NPB rice seedlings were selected. Whole seedlings were completely submerged in either 100 nM flg22 or 8 nM chitin solution. Triplicate samples of each treatment group were established, with untreated plants at time zero serving as the control. At intervals of 0.5-, 1-, 3- and 6-hours post-treatment (hpt), the seedlings were swiftly removed from the solution. Immediately thereafter, the plants were flash-frozen in liquid nitrogen and stored in a -80 °C ultra-low temperature freezer. Subsequently, RNA was extracted, reverse transcribed, and RT-qPCR was conducted to assess the expression levels of genes associated with the plant’s PTI pathway. Data were analyzed by Student’s *t*-test using SPSS, with significance set at * *P* < 0.05 and ** *P* < 0.01. Each treatment was performed in triplicate, both technically and biologically, to ensure reliability and reproducibility.

### 2.5 MeJA and SA treatment

Hormone treatments were carried out with minor modifications to previously described methods (Peng et al., 2021a). For hormone treatments, three-week-old rice seedlings were sprayed with either 2 mM salicylic acid (SA) or 100 µM methyl jasmonate (MeJA). Following treatment, the seedlings were kept in an incubator under conditions of constant humidity and temperature to ensure uniform environmental exposure. Sampling was carried out at 0, 2, 6, and 12 hpt for subsequent reverse transcription RT-qPCR analysis. Data were analyzed by Student’s *t*-test using SPSS, with significance set at * *P* < 0.05 and ** *P* < 0.01. Each treatment included three independent biological replicates; each measured in technical triplicate.

### 2.6 ROS detection

Reactive oxygen species (ROS) detection was performed as previously described (You et al., 2023). Leaves from one-month-old rice plants were punched to create discs with a diameter of 4.5 cm and then soaked in sterilized water overnight. After removing the excess water with paper towels, two leaf discs were placed in a 1.5 mL centrifuge tube containing a mixture of 98 μL Immun-star reagent, 1 μL horseradish peroxidase, and 8 nM chitin. Luminescence, indicative of ROS activity, was recorded at 1-minute intervals for a duration of 20 minutes using a Glomax Single-Tube Luminometer system (Promega). Each treatment was conducted in triplicate, both technically and biologically, to ensure the reliability and reproducibility of the results.

### 2.7 Transactivation activity assays in yeast

For the assessment of transactivation activity, the full-length and truncated coding sequences of *OsWHY1* were cloned into the pGBKT7 vector. The resulting constructs were then transformed into the yeast strain AH109 utilizing the lithium acetate/salicylic acid/polyethylene glycol (LiAc/SS-DNA/PEG) method, a standard technique for yeast transformation. The transformed yeasts were uniformly spread on synthetic dropout (SD) auxotrophic yeast media lacking tryptophan (SD/-Trp) or additionally lacking tryptophan, histidine, and adenine (SD/-Trp/-His/-Ade) to select for yeast containing the respective plasmids. The plates were incubated at 30 °C for 4 days to allow for growth, thereby indicating the presence of transactivation activity.

### 2.8 Y2H assay

The full-length *OsWHY1* fragment was cloned into both the activation domain (AD) vector (pGADT7) and the binding domain (BD) vector (pGBKT7). These constructs were utilized to co-transform the yeast strain AH109. The co-transformed yeasts were uniformly spread on synthetic dropout (SD) auxotrophic yeast media lacking leucine and tryptophan (SD/-Leu/-Trp) or additionally lacking histidine (SD/-Leu/-Trp/-His) to select for successful co-transformants. The plates were incubated at 30 °C for a period of 4 days to allow for growth and colony formation.

### 2.9 BiFC assay

The full-length *OsWHY1* fragment was subcloned at the N-terminus of the pSPYNE(R)173 vector and at the C-terminus of the pSPYCE(M) vector, resulting in the constructs pSPYNE(R)173-*OsWHY1* and pSPYCE(M)-*OsWHY1*. *Agrobacterium* strain EHA105, transformed with these constructs along with the viral suppressor p19 construct, was mixed in equal proportions and used for agroinfiltration into *N. benthamiana* leaves. The transfected leaves were subsequently examined using an inverted fluorescence microscope 48 hours post-infiltration to assess the bioluminescence resulting from the BiFC assay.

### 2.10 RNA-seq analysis

Three-week-old NPB and *OsWHY1*-overexpressing plants were inoculated with *M. oryzae*, each comprising pooled leaf tissue from 5 plants, were subjected to RNA sequencing (RNA-seq) analysis. The sequencing was conducted on the Illumina NovaSeqTM 6000 platform by LC Bio Technology Co., Ltd (Hangzhou, China). We extend our gratitude to LC Bio Technology Co., Ltd for their expertise in sequencing and bioinformatics analysis. The bioinformatic analysis was facilitated using the OmicStudio tools, which are accessible at https://www.omicstudio.cn/tool. The heatmap representation of the RNA-seq data was generated utilizing the R programming language, available at https://www.r-project.org/, integrated within the OmicStudio platform at https://www.omicstudio.cn/tool.

### 2.11 Gene accession numbers

Gene information in this article can be found in Rice Genome Annotation Project database (http://rice.uga.edu/) with the following accession numbers: *OsWHY1* (Os06g0145800), *OsPR2* (Os01g0940700), *OsWAK1* (Os11g0690066), *OsWRKY53* (Os05g0343400), *OsHSP58.7* (Os09g0491772), *OsMYB1R* (Os02g0685200), *OsSAG12* (Os04g0208200), *OsCATB* (Os06g0727200), and *OsUBQ* (Os03g0234200), The *MoPot2* accession number can be found in the National Center for Biotechnology Information as Z33638.

## 3 Results

### 3.1 *OsWHY1* expression was induced by exogenous hormones, PAMPs and *M. oryzae* treatment.

In this study, we examined the expression of OsWHY1 in response to treatment with exogenous hormones (MeJA and SA). The results showed that MeJA markedly induced the expression of *OsWHY1* at 2 hpt (**Figure 1a**). However, after SA treatment, *OsWHY1* was significantly down-regulated at 2 hpt (**Figure 1a**). As shown in **Figure 1b**, the transcript level of *OsWHY1* increased rapidly upon perception of flg22 with a peaking at 3 hpt. Interestingly, *OsWHY1* expression was inhibited after chitin treatment at 0.5 hpt, and expression levels reached their lowest after 3 hpt. The contrasting responses to SA and MeJA, as well as to flg22 and chitin, raise the possibility that OsWHY1 contributes to the regulation of PTI-related responses.

**Figure 1:**
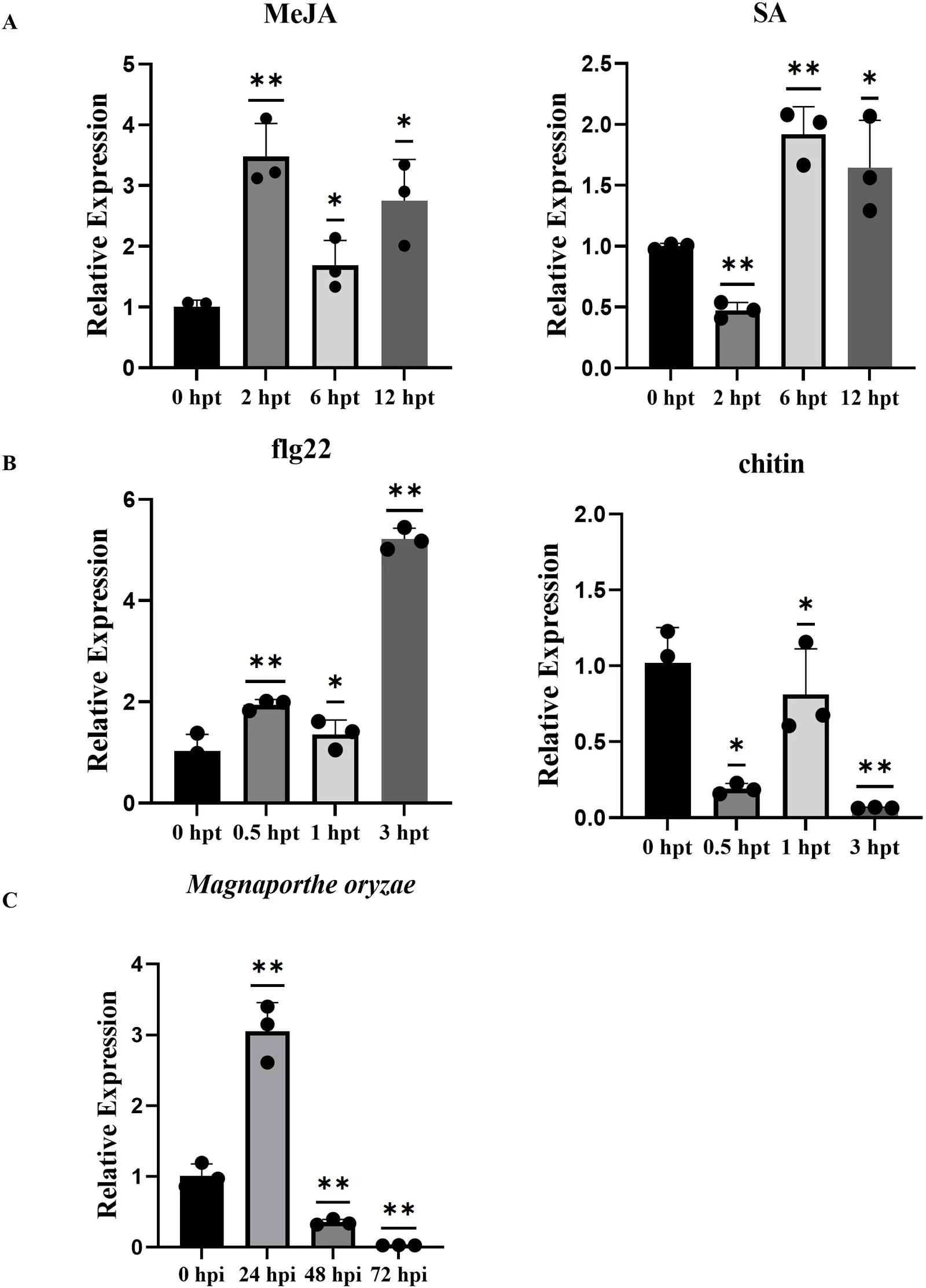
Relative expression of *OsWHY1* in Nipponbare (NPB) after methyl jasmonate (MeJA), salicylic acid (SA), chitin, flg22, *M. oryzae* treatment. Three-week-old NPB rice plants were exposed to different treatments: **(A)** 2 nM salicylic acid (SA), 100 μM methyl jasmonate (MeJA); **(B)** 100 nM flg22, 8 nM chitin; and **(C)** inoculation with *M. oryzae*. Leaf samples were collected at specified time points post-treatment for analysis. The transcript levels of the *OsWHY1* gene were quantified using reverse transcription RT-qPCR and normalized to the endogenous control gene *OsUBQ*. The data are presented as means ± standard error (SE), derived from three independent experiments. Error bars indicate the standard error of the mean (SEM). Statistical comparisons were made using Student’s t-tests to assess the significance of expression changes relative to the 0-hour time point for each treatment: *P < 0.05, **P < 0.01.

To elucidate the role of *OsWHY1* in rice immunity, we quantified its transcript levels at various time points following infection with *M. oryzae*. The expression profile of *OsWHY1* exhibited an initial surge, with a threefold increase observed at 24 hpi, as illustrated in **Figure 1c**. Subsequently, the expression levels began to decline, reaching their nadir at 72 hpi. This pattern suggests that OsWHY1 may be involved in regulating the rice immune response to *M. oryzae*.

### 3.2 OsWHY1 negatively regulates plant defense against *M. oryzae.*

We generated transgenic rice plants overexpressing *OsWHY1* as well as CRISPR-Cas9-mediated knockout mutants in the genetic backgrounds of the cultivars NPB and Zhonghua11. Among 12 independent T0 lines, three independent mutant lines (*oswhy1-1*, *oswhy1-12*, and *oswhy1-17*) were identified. *oswhy1–1* carried a 1-bp insertion at position 260 bp, *oswhy1–12* carried a 2-bp deletion at positions 71–72 bp, and *oswhy1–17* carried a 1-bp insertion at position 80 bp. All three lines exhibited biallelic mutations predicted to cause frameshifts and premature stop codons. Sequencing chromatograms are shown in **Supplementary Figure S1a**. In addition, the knockout of OsWHY1 resulted in albino seedlings and subsequent lethality (**Supplementary Figure S1b**), suggesting an essential role for OsWHY1 in chloroplast development. Consequently, our study focused on the overexpressing *OsWHY1* lines to investigate its function in rice immunity. A total of twenty-eight *OsWHY1* overexpression lines were obtained, with lines 4 and 18 emerging as the most representative due to their notably higher expression levels (**Supplementary Figure S1c**).

To elucidate the role of OsWHY1 in rice immunity, we assessed disease symptoms in both the NPB control and *OsWHY1*-overexpressing plants following infection by *M. oryzae*. The overexpressing plants displayed significantly larger lesion areas and a higher number of lesions compared to NPB plants after spray-inoculation with *M. oryzae* isolate 110-2 (**Figure 2a**). Furthermore, the relative fungal biomass in the leaves of *OsWHY1*-OX-4 and *OsWHY1*-OX-18 plants was approximately six and five times higher, respectively, than in NPB leaves following spray-inoculation with *M. oryzae* (*P* < 0.01, **Figure 2b**). To validate the infection phenotypes observed with the spray-inoculation method, we also conducted punch-inoculation with *M. oryzae*. The results confirmed that the lesion size was significantly larger in the *OsWHY1*-overexpressing plants than in NPB (**Figure 2c**). Upon measuring the lesion length, we found that *OsWHY1*-OX-4 and *OsWHY1*-OX-18 had lesions that were 1.4 and 1.7 times larger, respectively, than those in NPB (*P* < 0.05, **Figure 2d**). Collectively, these findings indicate that OsWHY1 acts as a negative regulator of rice immunity against *M. oryzae*.

**Figure 2:**
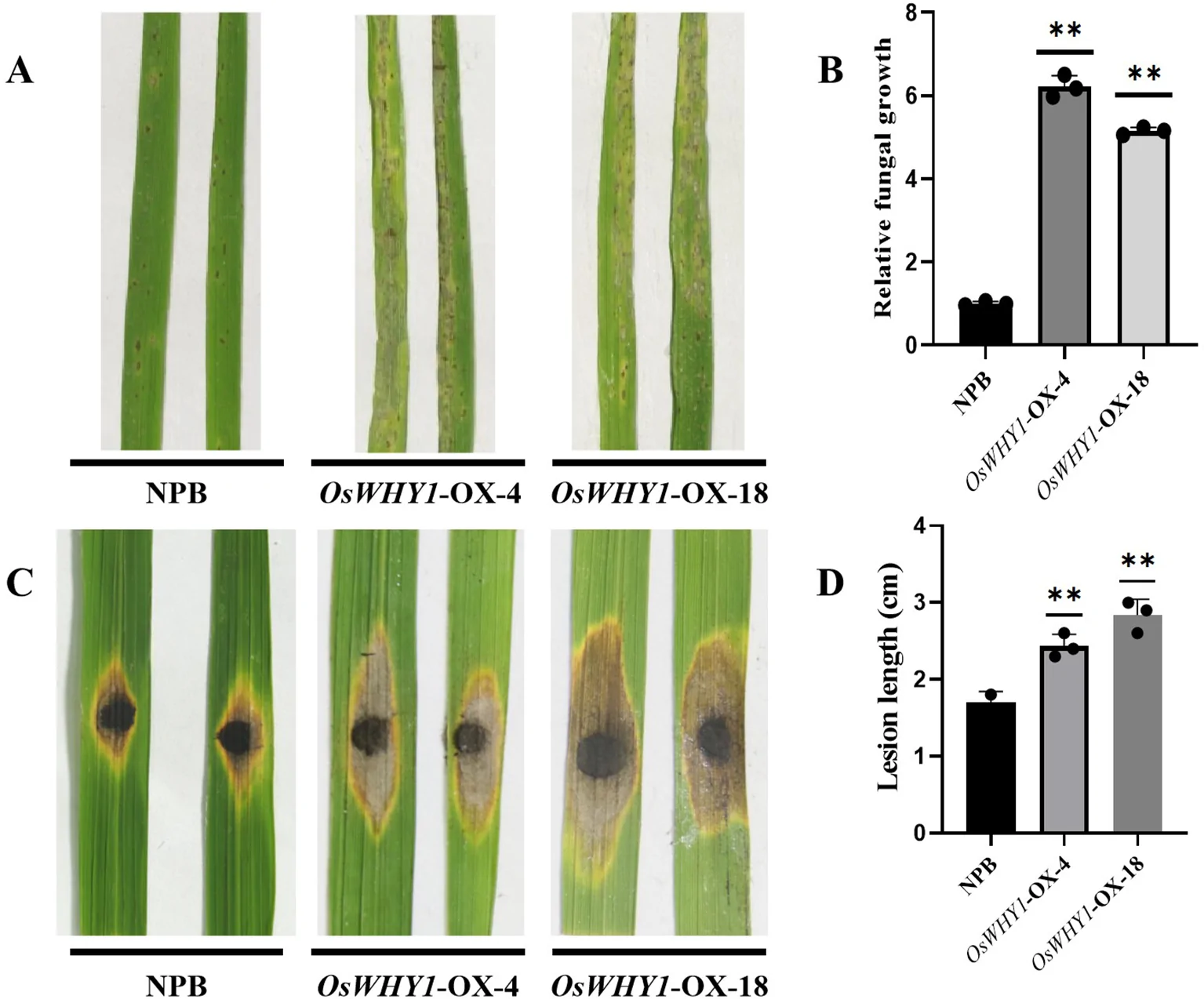
*OsWHY1* negatively regulates rice immunity against *Magnaporthe oryzae*. Leaves of Nipponbare (NPB) and *OsWHY1*-OX rice plants were inoculated with 5 μL of *M. oryzae* spore suspension, and photographs of the inoculated areas were captured at 5 days post-inoculation (dpi). Panel **(A)** displays disease symptoms following spray inoculation, **(B)** illustrates relative fungal growth, **(C)** shows disease symptoms after punch inoculation, and **(D)** presents the lesion length measurements on representative leaves from both *OsWHY1*-OX and NPB plants. Relative fungal growth was determined by quantifying the ratio of the *M. oryzae*-specific *MoPot2* gene level to the constitutive rice *OsUBQ* gene using quantitative RT-qPCR. Statistical significance between the groups was assessed using Student’s t-tests, with * indicating *P* < 0.05 and ** indicating *P* < 0.01.

### 3.3 Transcriptional activity analysis of OsWHY1.

To evaluate the transcriptional activity of OsWHY1, we constructed a fusion plasmid, pGBKT7-OsWHY1, and compared it with the negative control, pGBKT7, by transforming them into the yeast strain AH109. Yeast cells harboring the pGBKT7-OsWHY1 plasmid failed to grow on synthetic dropout (SD) medium lacking tryptophan, histidine, and adenine (-Trp/-His/-Ade), indicating a lack of transcriptional activation. Additionally, the deletion mutants pGBKT7-OsWHY1-N (amino acids 1-97, lacking the conserved Whirly domain) and pGBKT7-OsWHY1-C (amino acids 98-272, lacking the entire N-terminus) also failed to activate the transcription of the reporter gene (**Figure 3a**). These findings suggest that OsWHY1 lacks intrinsic transcriptional activation capacity.

**Figure 3:**
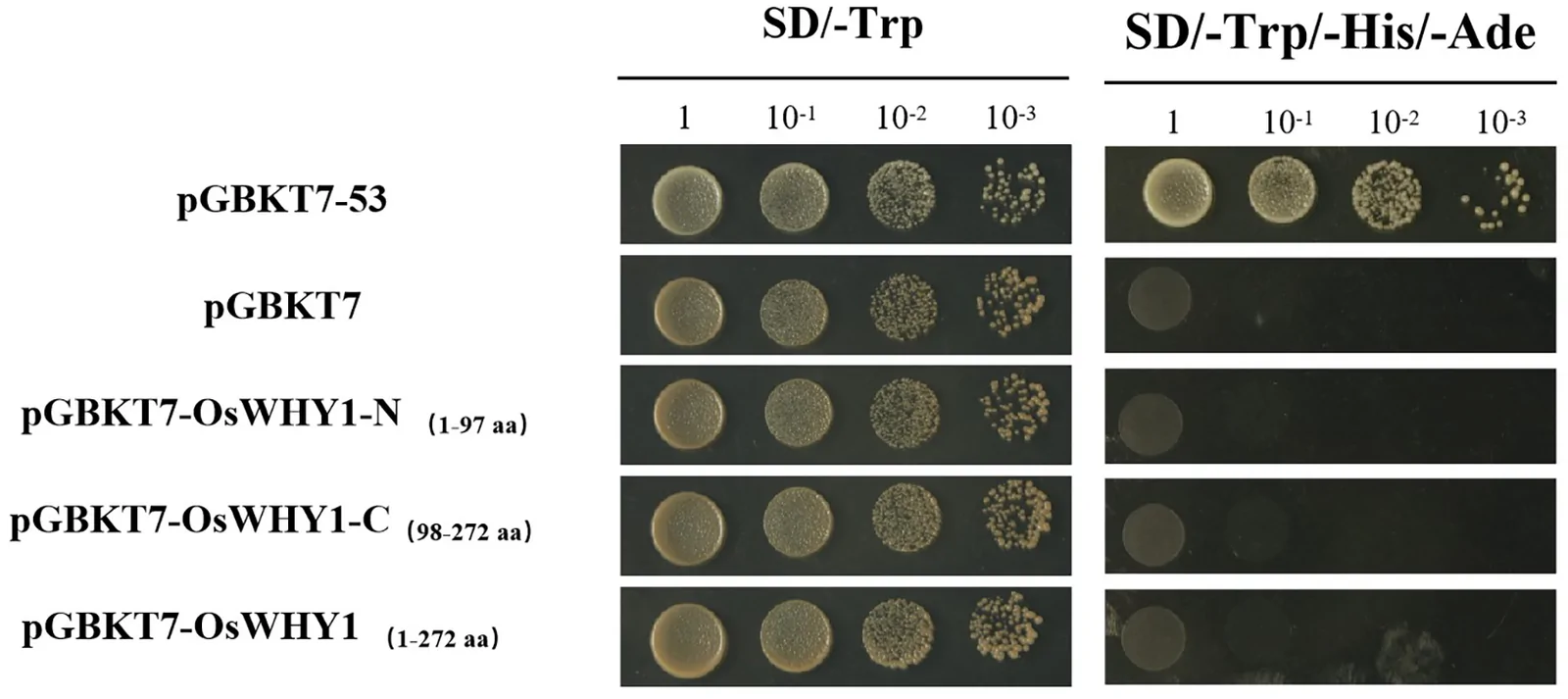
OsWHY1 not possesses transcriptional activation activity. The transactivation activity of OsWHY1 and its deletion variants in yeast two-hybrid assays.

### 3.4 Homomeric interactions in OsWHY1 proteins.

Based on previous studies, PBF-2 has been characterized as a p24 tetramer with a distinct structure (Despres et al., 1995). In this study, we explored the potential for OsWHY1 to form homomeric interactions using yeast two-hybrid assays. As depicted in **Figure 4a**, yeast cells co-transformed with the pGBKT7-OsWHY1 and pGADT7-OsWHY1 vectors were able to grow on synthetic dropout (SD) medium lacking tryptophan, histidine, and leucine (-Trp/-His/-Leu). This growth indicates that OsWHY1 is capable of homomeric interaction within the yeast cells. Intriguingly, when the deletion mutants pGBKT7-OsWHY1-N and pGBKT7-OsWHY1-C were co-transformed with pGADT7-OsWHY1 into yeast cells, growth was not observed on the selective medium, suggesting that the structural integrity necessary for homomeric interaction is compromised by these deletions. These findings collectively suggest that OsWHY1 can engage in homomeric interactions *in vivo* within yeast cells, and that the integrity of the full-length protein is crucial for this interaction.

**Figure 4:**
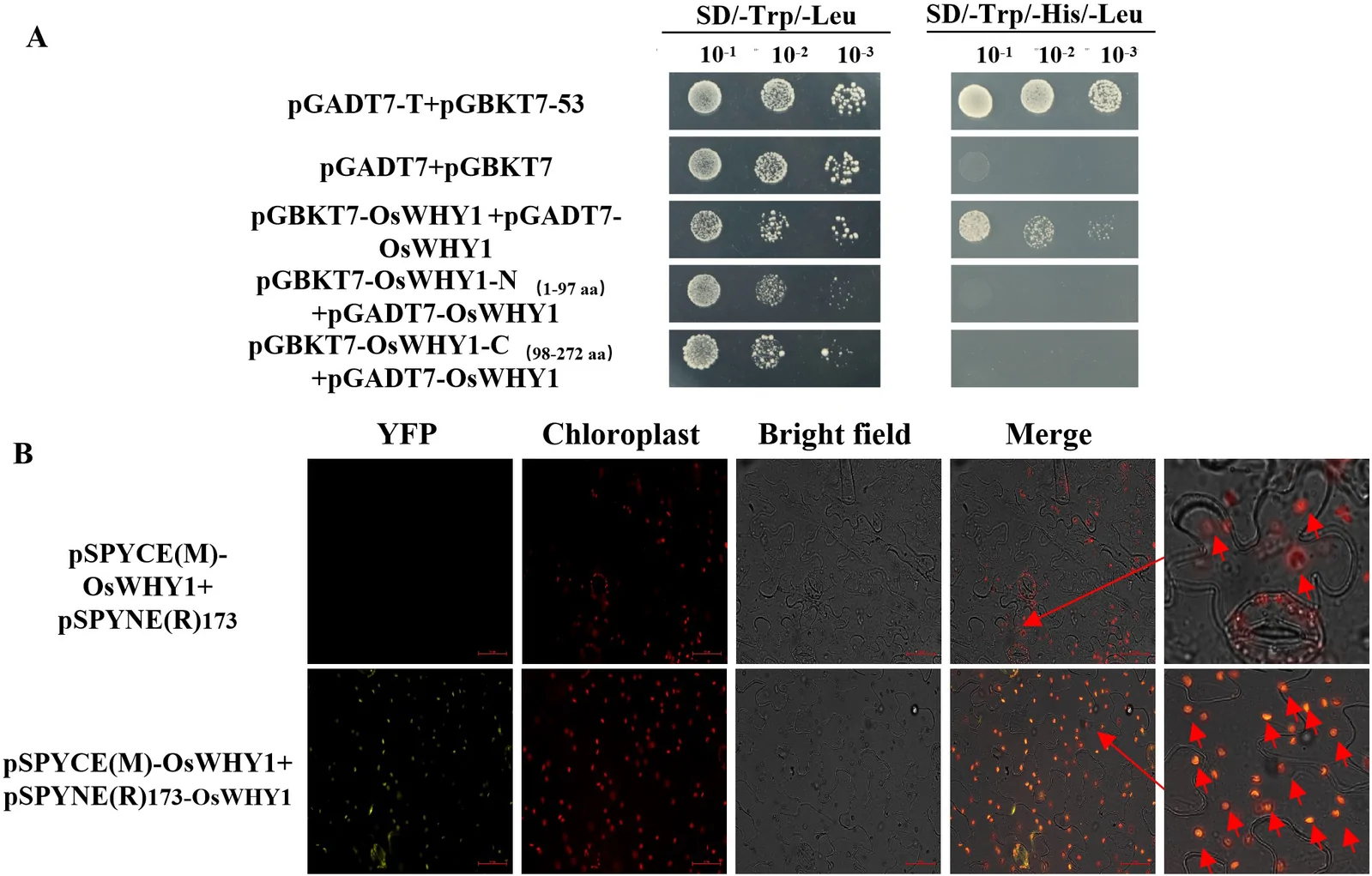
The interaction assays confirming the homomeric interaction of OsWHY1. **(A)** Yeast two-hybrid (Y2H) assay demonstrating the self-interaction of OsWHY1. **(B)** Bimolecular fluorescence complementation (BiFC) assay providing further evidence of the direct homomeric interaction of OsWHY1. For the BiFC assay, Agrobacterium strains carrying complementary plasmids pSPYNE(R)173-OsWHY1 and pSPYCE(M)-OsWHY1 were infiltrated into *Nicotiana benthamiana* leaves. Yellow fluorescent protein (YFP) signals, indicative of successful interaction, were visualized under a confocal laser scanning microscope 48 hours post-infiltration. The images captured include dark field views for YFP and chloroplast autofluorescence, bright field views for cell morphology, and merged images to illustrate the co-localization. Scale bars represent 50 μm.

To further ascertain the potential for OsWHY1 to form homomeric structures, we conducted bimolecular fluorescence complementation (BiFC) assays. As illustrated in **Figure 4b**, robust yellow fluorescent protein (YFP) activity was observed within the chloroplasts when the constructs pSPYCE(M)-OsWHY1 and pSPYNE(R)173-OsWHY1 were co-expressed. This YFP activity merged with the intrinsic red fluorescence of the chloroplasts, resulting in an orange fluorescence signal. Conversely, when pSPYCE(M)-OsWHY1 was co-transformed with the pSPYNE(R)173 empty vector into *Nicotiana benthamiana* as a negative control, no fluorescence was detected. These results provide compelling evidence that OsWHY1 is capable of forming homomeric interactions within the chloroplasts.

### 3.5 Transcriptome analysis of the *OsWHY1*- overexpressing plants.

To gain a deeper understanding of the role of *OsWHY1* in mediating rice transcriptional regulation during plant immune responses, we conducted a comprehensive transcriptome analysis comparing the gene expression profiles of NPB and *OsWHY1*- overexpressing plants at 24 hpi with *M. oryzae*. Our analysis revealed a significant disparity in gene expression between the two groups, with 296 differentially expressed genes (DEGs) identified-132 of which were down-regulated and 164 up-regulated in the *OsWHY1*- overexpressing plants, relative to NPB (**Supplementary Figure S2**). These DEGs exhibited a Log_2_ Fold change of >= 2 and *P* value of < 0.05, indicating a statistically significant alteration in expression levels.

To uncover the biological functions associated with the DEGs in the *OsWHY1*- overexpressing plants, we utilized Gene Ontology (GO) annotation analysis. The most enriched GO categories among the DEGs in the up- and down-regulated groups were identified as ‘regulation of DNA-templated transcription’ (GO:0006355) and ‘defense response’ (GO:0006952), respectively (**Figure 5a**). To visually represent the impact on specific genes, heat maps were constructed for several significantly down-regulated genes (**Figure 5b**). The down-regulated genes included a variety of stress-responsive genes, members of the *WRKY*, heat shock protein (HSP), and *NAC* families, as well as genes related to glutathione S-transferases (GSTs), which were predominantly affected (**Figure 5b**).

**Figure 5:**
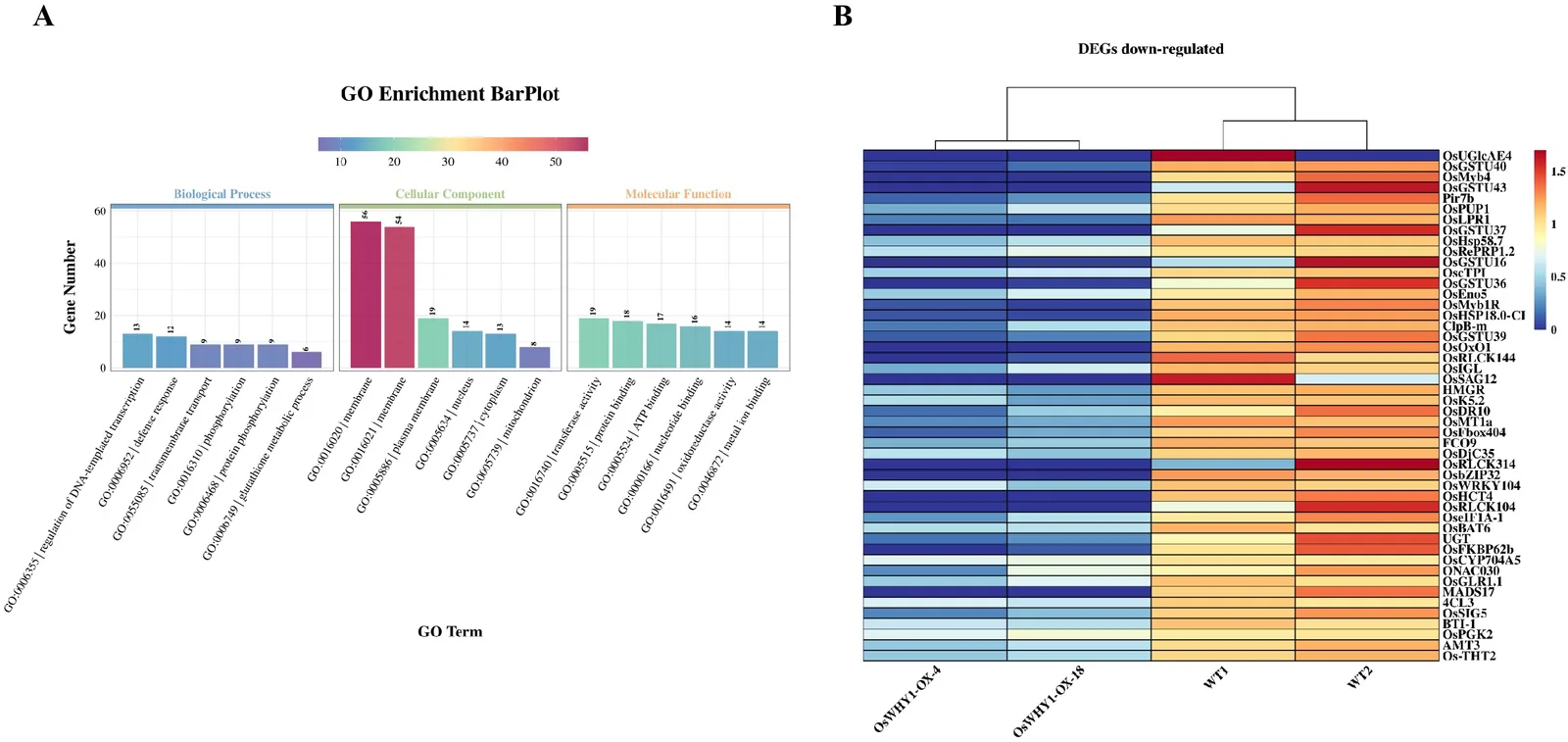
Gene ontology (GO) enrichment and expression analysis of differentially expressed genes (DEGs) in *OsWHY1*-overexpressing and Nipponbare (NPB) rice plants infected with *Magnaporthe oryzae*. **(A)** Graphical representation of enriched Gene Ontology **(GO)** terms associated with the differentially expressed genes identified in *OsWHY1*-OX compared to NPB plants following infection with *M. oryzae* at 24 hours post-inoculation (hpi). **(B)** Heatmap illustrating the expression patterns of DEGs that were downregulated in *OsWHY1*-OX plants relative to NPB controls under the same conditions. The color scale indicates the relative expression levels, with red representing higher expression and blue representing lower expression. The heatmap provides a visual summary of the transcriptional changes that may contribute to the differential response to *M. oryzae* infection in *OsWHY1*-OX and NPB plants.

### 3.6 OsWHY1 negatively regulates rice immunity against *M. oryzae*-induced and chitin-triggered PTI.

To elucidate the mechanism by which OsWHY1 modulates rice immunity, we quantified reactive oxygen species (ROS) accumulation in both *OsWHY1*-overexpressing and NPB control plants following chitin treatment. As shown in **Figure 6a**, the ROS burst induced by chitin was significantly attenuated in the *OsWHY1*-overexpressing plants compared to the NPB plants. These findings suggest that OsWHY1 may suppress the chitin-triggered ROS production in rice, potentially impacting the pathogen-associated molecular pattern-triggered immunity (PTI) pathway.

**Figure 6:**
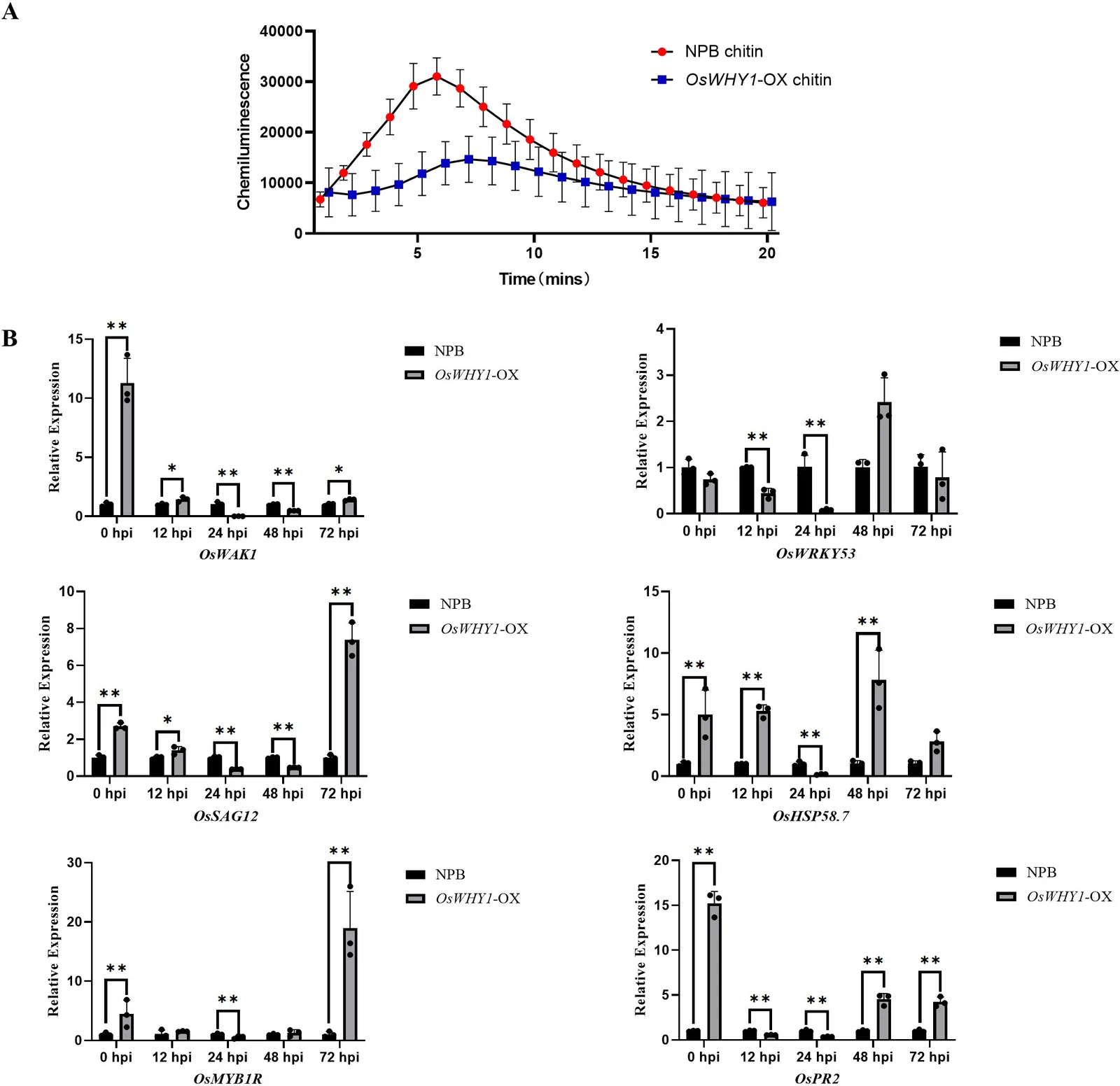
Chitin-induced reactive oxygen species (ROS) response and defense gene expression in *OsWHY1*-overexpressing rice plants. **(A)** Chitin-triggered ROS burst assay in leaf discs treated with 8 nM chitin. The chemiluminescent signal, indicative of ROS production, was continuously monitored and the results are expressed as relative luminescence units. **(B)** Relative expression levels of defense and signaling genes in *OsWHY1*-OX plants under normal conditions and upon infection with *Magnaporthe oryzae*. Statistical analysis was performed using Student’s t-tests to evaluate the significance of differences in gene expression between *M. oryzae*-treated NPB and *OsWHY1*-OX plants: *P < 0.05, **P < 0.01.

To delve deeper into the impact of *OsWHY1* overexpression on gene regulation, we selected several stress-related and PTI-related genes that were significantly downregulated in our transcriptome-wide analysis for validation. These included the stress-related genes *OsCATB*, *OsHSP58.7*, *OsMYB1R*, and *OsSAG12*, as well as the PTI-related genes *OsPR2*, *OsWAK1*, and *OsWRKY53*. We assessed the transcript levels of these PTI-related and stress-related genes at 24 hpi with *M. oryzae*. The results revealed that the expression of all tested genes was significantly downregulated in the *OsWHY1*-overexpressing plants relative to the NPB control plants (**Figure 6b**; **Supplementary Figure S4**). These RT-qPCR results are consistent with the transcriptome-wide trends, collectively suggesting that OsWHY1 exerts a negative regulatory effect on the expression of defense and signaling genes induced by *M. oryzae*.

## 4 Discussion

Whirly proteins, known for their DNA/RNA-binding capabilities, play a pivotal role in plant immunity. In this study, we discovered that OsWHY1 is inducible by both hormonal signals and pathogen-associated molecular patterns (PAMPs). Notably, expression profiling revealed a robust induction of OsWHY1 following inoculation with *M. oryzae*, particularly at 24 hpi. Furthermore, plants overexpressing *OsWHY1* displayed enhanced susceptibility to *M. oryzae* infection when compared to the NPB control. These findings indicate that OsWHY1 may function as a negative regulator of the rice response to *M. oryzae* infection. While the majority of disease resistance genes are induced upon pathogen challenge, a subset of susceptibility genes, such as *ONAC083*, a transcription factor, is also upregulated by pathogens. *ONAC083* has been shown to negatively regulate resistance to *M. oryzae* and to dampen chitin-induced PTI, and similar to OsWHY1, it is induced at 24 hpi with *M. oryzae* (Bi et al., 2023). The similar temporal induction of OsWHY1 and ONAC083 suggests that they might function in the same regulatory module to attenuate late-stage PTI responses, though whether they act independently or cooperatively remains to be determined. This similar induction pattern implies that OsWHY1 may function as part of a negative feedback mechanism to prevent excessive immune activation.

Having established OsWHY1 as a negative regulator, we next sought to uncover the transcriptional basis of its suppressive function. To further delineate the role of OsWHY1 in mediating rice transcriptional regulation during plant immune responses, we conducted a transcriptome analysis on NPB and *OsWHY1*- overexpressing plants following infection with *M. oryzae* at 24 hpi. The DEGs in the *OsWHY1*-overexpressing plants were significantly enriched in Gene Ontology (GO) categories related to ‘membrane’ and ‘transferase activity’. Among these, glutathione S-transferases (GSTs), an ancient protein superfamily with diverse functions, are crucial for the adaptation to both abiotic and biotic stresses. They contribute to the modulation of cellular redox status, the biosynthesis, and the transport of secondary metabolites and hormones (Nianiou-Obeidat et al., 2017; Vaish et al., 2020). We identified six *GST* genes (*OsGSTU16*, *OsGSTU36*, *OsGSTU37*, *OsGSTU39*, *OsGSTU40*, and *OsGSTU43*) that were significantly downregulated in the *OsWHY1*-overexpressing plants. This suggests that OsWHY1 may be implicated in the glutathione S-transferase activity pathway, potentially influencing the plant’s response to stress and pathogens. The downregulation of *GST* transcripts may suggest a potential reduction in the plant’s capacity for ROS detoxification and hormonal balance, which could contribute to weakened defense signaling and increased susceptibility to *M. oryzae*. However, since GST enzymatic activity was not directly measured in this study, this interpretation remains tentative and requires future biochemical validation. Given the DNA-binding capacity of OsWHY1, it is conceivable that OsWHY1 may associate with the promoters of *GST* genes and thereby influence their transcriptional regulation; however, direct experimental evidence for such promoter binding is currently lacking, and alternative indirect mechanisms cannot be ruled out. Future experiments such as ChIP-qPCR or EMSA will be needed to test this possibility. Beyond transcriptional changes, the ROS burst is a hallmark of PTI and a critical readout of immune execution. The plant immune response is characterized by a series of signaling cascades, including ROS burst, which is a pivotal event in the interaction between rice and the pathogen *M. oryzae*. In this study, we observed that the ROS response in *OsWHY1*-overexpressing plants was diminished compared to the NPB control following chitin treatment. To further substantiate the role of OsWHY1 in the disease resistance response and to validate our transcriptome data, we examined the expression of a series of downstream genes associated with the disease resistance response in the *OsWHY1* overexpressing plants. These genes were selected based on their significant downregulation in our transcriptome-wide analysis and their established roles in PTI and stress signaling. This included genes such as *OsWAK1, OsPR2*, and *OsWRKY53*, which are well-established components of the defense’s mechanism (Chujo et al., 2009; Li et al., 2009; Chujo et al., 2014; Hu et al., 2016). Additionally, we analyzed stress-related genes identified, including *OsCATB*, *OsHSP58.7*, *OsMYB1R*, and *OsSAG12*, which are known to be involved in stress responses (Dubos et al., 2010; Singh et al., 2013; Ni et al., 2022; Fortunato et al., 2023). The expression of these selected genes was found to be downregulated in the *OsWHY1*-overexpressing plants at 24 hpi compared to NPB. OsWHY1 may contribute to ROS homeostasis by regulating genes involved in ROS production or scavenging, and the impaired ROS burst likely compromises downstream PTI signaling. Given that chloroplasts are a major source of ROS production, this chloroplast-related regulatory function is consistent with the known role of OsWHY1 in chloroplast development. Indeed, our observation that complete loss of OsWHY1 causes albino and lethal phenotypes agrees with Qiu et al. (2022), who showed that OsWHY1 participates in the processing of chloroplast-related gene RNAs, thereby regulating chloroplast development. Therefore, OsWHY1 likely exerts additional functions within the chloroplast to modulate immunity. Although OsWHY1 forms homomeric complexes in chloroplasts, this localization does not necessarily require its translocation to the nucleus for regulatory function. Instead, OsWHY1 may act within the chloroplast to regulate *GST* family genes, since several GST members are also localized in chloroplasts and participate in redox regulation and detoxification (Vaish et al., 2020). For nuclear defense-related genes, OsWHY1 may exert its effects indirectly by modulating chloroplast-derived retrograde signaling components, rather than by directly binding to their promoters in the nucleus. Collectively, our results show that OsWHY1 overexpression is associated with reduced expression of defense-related genes and diminished chitin-induced ROS accumulation. These associations could be consistent with a possible negative regulatory role for OsWHY1 in rice immunity; however, they do not yet establish a direct causal link between GST repression and the increased susceptibility phenotype. Additional genetic or biochemical evidence would be needed to clarify this relationship.

The repressive function of OsWHY1 may be rooted in its unique structural features compared to other WHIRLY proteins. WHIRLY proteins are characterized by a highly conserved WHIRLY DNA-binding domain that is essential and sufficient for effective single-stranded DNA (ssDNA) binding. The relatively conserved Whirly domain of WHY1 proteins is known to form homomeric structures that facilitate their DNA-binding function and transcriptional regulation (Desveaux et al., 2002; Taylor et al., 2023). In this study, we found that the OsWHY1 protein, like other WHY1 proteins, forms homomeric structures, suggesting that it possesses the fundamental structural characteristics of a WHY1 protein, which is likely required for its DNA-binding function. However, most WHY1 proteins identified in other plants function as positive regulators of plant defenses, such as *ZmWHY1* and *CsWHY1* (Kretschmer et al., 2017; Yan et al., 2020). Phylogenetic analysis indicates that OsWHY1 has diverged significantly from other WHY1 proteins (**Supplementary Figure S3**) (Lai et al., 2022), with key differences located in the N-terminal activation domain (Krupinska et al., 2022). For instance, the N-terminal region of StWhy1, which contains a polyglutamine stretch, acts as a transactivation domain, and similar domains are present in the tomato LeWhy1 and grapevine VvWhy1 (Desveaux et al., 2005). In our study, we assessed the transcriptional activation capacity of OsWHY1 and found that it lacks such activity. Although direct experimental validation is still required, we hypothesize that the absence of transcriptional activation in OsWHY1 is due to variations in its N-terminal activation domain. Moreover, as OsWHY1 lacks intrinsic transactivation activity, it likely requires interaction with other transcriptional regulators to exert its repressive effect on defense genes. This is reminiscent of the regulation of MYC proteins by the JAZ3 protein in the JA signaling pathway, where interaction with the MYC N-terminus results in the loss of transcriptional activity, thereby affecting downstream gene expression (Chini et al., 2007). We therefore propose that significant changes in the domain structure outside the conserved region of OsWHY1 may underlie its role as a negative regulator of rice defense’s mechanisms. Thus, OsWHY1 represents an unusual negative regulator within the WHIRLY family, likely due to its lack of transactivation activity, distinguishing it from canonical WHY1 activators. We recognize that the lethality of homozygous *oswhy1* mutants limits our functional analysis to overexpression lines, and therefore our conclusions regarding the immune function of OsWHY1 should be interpreted with appropriate caution. Given that complete loss of *OsWHY1* causes a severe albino/lethal phenotype, future studies using conditional, tissue-specific, or inducible knockdown systems will be essential to dissect the role of OsWHY1 in immunity independently of its function in chloroplast development.

## 5 Conclusions

In summary, our study demonstrates that the expression of *OsWHY1* is induced by exogenous hormones, PAMPs, and infection with *M. oryzae*. Although OsWHY1 is capable of forming homomeric structures, it lacks a transcriptional activation function, which distinguishes it from most characterized dicot WHY1 proteins that function as positive immune regulators. We found that OsWHY1 functions as a negative regulator of rice resistance to *M. oryzae*, exerting its effects by suppressing the expression of key defense genes and the accumulation of ROS. These findings reveal a novel negative regulatory role for a monocot WHIRLY protein and provide a valuable framework for future studies. Immediate next steps include testing whether OsWHY1 directly binds to the promoters of defense-related genes (e.g., GSTs) and performing domain-swap experiments with a dicot activator WHY1 to elucidate the structural basis of this functional divergence.

## Data Availability

The datasets presented in this study can be found in online repositories. The names of the repository/repositories and accession number(s) can be found below: https://www.ncbi.nlm.nih.gov/, GSE270244.
