# Supplementary material for: OsWHY1 Negatively Modulates Rice Immunity Against Magnaporthe oryzae
Source: Front Plant Sci. 2026 Sep 28;17:1931455. doi: 10.3389/fpls.2026.1931455 (PMC13655234; doi:10.3389/fpls.2026.1931455)
Supplement: Supplementary file 1 [file DataSheet1.docx]

Supplementary Material

**Figure S1**

**Figure S1. Identification of *oswhy1* plants and *OsWHY1*-OX plants.**

(A) Representative sequences of mutant allele identified from a single plant of the *oswh1* mutant. (B) Growth phenotype of the *oswhy1* mutant rice plants, exhibiting distinct characteristics compared to the wild-type (ZH11). (C) Expression levels of the *OsWHY1* gene in *OsWHY1*-overexpressing (OX) plants, as determined by quantitative real-time polymerase chain reaction (qRT-PCR). The data are presented as the mean ± standard deviation (SD). Statistical significance was assessed using Student's t-tests, comparing the expression levels of *OsWHY1* in OX plants to the control: * *P* < 0.05, ** *P* < 0.01.

**Figure S2**

**Figure S2. Differentially expressed genes in transcriptome analysis.**

A volcano plot illustrates the differentially expressed genes (DEGs) in *OsWHY1*-overexpressed plants relative to the Nipponbare control, highlighting the significant changes in gene expression (Log2 Fold change ≥ 2, *P* value < 0.05).

**Figure S3**

**Figure S3. phylogenetic tree analysis.**

Schematic representation of conserved motifs and amino acid sequence comparison among selected WHIRLY proteins from various plant species. The accession numbers for each protein are as follows: OsWHY1 (*Oryza sativa*, XP_015641336.1), PhWHY1 (*Panicum hallii*, XP_025810386.1), ZmWHY1 (*Zea mays*, NP_001123589.1), PvWHY1 (*Panicum virgatum*, XP_039806747.1), TaWHY1 (*Triticum aestivum*, XP_044426484.1), PmWHY1 (*Panicum miliaceum*, RLM56178.1), HvWHY1 (*Hordeum vulgare*, KAE8776928.1), GmWHY1 (*Glycine max*, XP_014634831.1), NtWHY1 (*Nicotiana tabacum*, XP_016474450.1), SlWHY1 (*Solanum lycopersicum*, AFY24240.1), CaWHY1 (*Capsicum annuum*, XP_016548546.1), VvWHY1 (*Vitis vinifera*, XP_002277278.1), CpWHY1 (*Carica papaya*, XP_021897420.1), StWHY1 (*Solanum tuberosum*, NP_001275155.1), AtWHY1 (*Arabidopsis thaliana*, NP_172893.1). The motifs were identified using the MEME suite.

**Figure S4**

**Figure S4. Relative expression levels of defense and signaling genes.**

Relative expression levels of defense and signaling genes in *OsWHY1*-OX plants under normal conditions and upon infection with *M. oryzae*. Statistical analysis was performed using Student's t-tests to evaluate the significance of differences in gene expression between *M. oryzae*-treated NPB and *OsWHY1*-OX plants: * *P* < 0.05, ** *P* < 0.01.
