# Supplementary material for: OsWHY1 Negatively Modulates Rice Immunity Against Magnaporthe oryzae
Source: Front Plant Sci. 2026 Sep 28;17:1931455. doi: 10.3389/fpls.2026.1931455 (PMC13655234; doi:10.3389/fpls.2026.1931455)
Supplement: Supplementary file 2 [file Table1.docx]

| **Table S1 Primers for PCR and RT-qPCR** | | | | | |  |  |
| --- | --- | --- | --- | --- | --- | --- | --- |
|  | **Abbreviation** | **ID** | **Sequence (5’ to 3’)** | **Amplicon size** |  |  |  |
|  | OsWHY1-F | Os06g0145800 | ATGCCGCCACCGTCG | 816 bp |  |  |  |
|  | OsWHY1-R |  | CCTCCGCCATTCATATTCAGG |  |  |  |  |
|  | OsWHY1-pGBKT7-N _(1-97aa)_-F |  | CATATGGCCATGGAGGCCGAATTCATGCCGCCACCGTCG | 338 bp |  |  |  |
|  | OsWHY1-pGBKT7-N _(1-97aa)_-R |  | TGCTAGTTATGCGGCCGCTGCAGGCTGTACGTAGAGAACACCCTCC |  |  |  |  |
|  | OsWHY1-pGBKT7-C _(98-272aa)_-F |  | CATATGGCCATGGAGGCCATCTACAAGGGCAAGGCGG | 572 bp |  |  |  |
|  | OsWHY1-pGBKT7-C _(98-272aa)_-R |  | TGCTAGTTATGCGGCCGCTGCAGGTCGACCCTCCGCCATTCATATTCAGG |  |  |  |  |
|  | OsWHY1-pGBKT7-F |  | CATATGGCCATGGAGGCCGAATTCATGCCGCCACCGTCG | 869 bp |  |  |  |
|  | OsWHY1-pGBKT7-R |  | TGCTAGTTATGCGGCCGCTGCAGGTCGACCCTCCGCCATTCATATTCAGG |  |  |  |  |
|  | OsWHY1-pGADT7-F |  | GCTCATATGGCCATGGAGGCCAGTGAATTCATGCCGCCACCGTCG | 878 bp |  |  |  |
|  | OsWHY1-pGADT7-R |  | CGATTCATCTGCAGCTCGAGCTCGATGGATCCCCTCCGCCATTCATATTCAGG |  |  |  |  |
|  | pSPYNE(R)173-OsWHY1-F |  | TGGGCCCAGGCCTACTAGTGGATCCATGCCGCCACCGTCG | 866 bp |  |  |  |
|  | pSPYNE(R)173-OsWHY1-R |  | CCTACCCGGGAGCGGTACCCTCGAG ACTCCTCCGCCATTCATATTCAGG |  |  |  |  |
|  | pSPYCE(M)-OsWHY1-F |  | GGCCTGGCGCGCCACTAGTGGATCCATGCCGCCACCGTCG | 866 bp |  |  |  |
|  | pSPYCE(M)-OsWHY1-R |  | ACATCCCGGGAGCGGTACCCTCGAGCCTCCGCCATTCATATTCAGG |  |  |  |  |
|  | UBQ-QF | Os03g0234200 | AACCAGCTGAGGCCCAAGA | 77 bp |  |  |  |
|  | UBQ-QR |  | ACGATTGATTTAACCAGTCCATGA |  |  |  |  |
|  | MoPot2-QF | Z33638 | ACGACCCGTCTTTACTTATTTGG | 99 bp |  |  |  |
|  | MoPot2-QR |  | AAGTAGCGTTGGTTTTGTTGGAT |  |  |  |  |
|  | OsWHY1-QF | Os06g0145800 | GGGATTCGTGCTGCTACAGT | 100 bp |  |  |  |
|  | OsWHY1-QR |  | GCTACCCATCTCCCACACAG |  |  |  |  |
|  | OsWAK1-QF | Os11g0690066 | GTGGTCCGTCTTTGGGGATT | 231 bp |  |  |  |
|  | OsWAK1-QR |  | TGGGCATCGTCAGACAACAA |  |  |  |  |
|  | OsSAG12-QF | Os04g0208200 | GACCAAGTACTGGCTGATGAAGA | 102 bp |  |  |  |
|  | OsSAG12-QR |  | CCACAGACGCCCTCCTTGTC |  |  |  |  |
|  | OsPR2-QF | Os01g0940700 | CTACGGCGTGATCGGGAACAAC | 114 bp |  |  |  |
|  | OsPR2-QR |  | GCCTGGAGGATGTCGCTTCTTG |  |  |  |  |
|  | OsWRKY53-QF | Os05g0343400 | CCTCCCATATCTTGGCGTCC | 188 bp |  |  |  |
|  | OsWRKY53-QR |  | TTGCTGCTCTTGCTCCTTGA |  |  |  |  |
|  | OsHSP58.7-QF | Os09g0491772 | GGAGATGGCCACAGACAACA | 232 bp |  |  |  |
|  | OsHSP58.7-QR |  | ATGTAGCTCAGCCTCCCTCA |  |  |  |  |
|  | OsMYB1R-QF | Os02g0685200 | ACGGCGACATGGATTTGTCA | 160 bp |  |  |  |
|  | OsMYB1R -QR |  | ATGTGCTCTTGTATACGGCG |  |  |  |  |
|  | OsCATB -QF | Os06g0727200 | GCCACGAAGGACTTGACTGA | 234 bp |  |  |  |
|  | OsCATB -QR |  | TATCGCTGGGCAGAAAGCAA |  |  |  |  |
