# Supplementary material for: OsWHY1 Negatively Modulates Rice Immunity Against Magnaporthe oryzae
Source: Front Plant Sci. 2026 Sep 28;17:1931455. doi: 10.3389/fpls.2026.1931455 (PMC13655234; doi:10.3389/fpls.2026.1931455)
Supplement: Supplementary file 3 [file Table2.docx]

**Table S2 Raw Ct values for all qRT-PCR experiments**

**MeJA treatment**

| **background** | **Abbreviation** | **Hours post-treatment** | **Raw Ct values** |
| --- | --- | --- | --- |
| NPB | *OsUBQ* | 0 h | 21.023655765 |
| NPB | *OsUBQ* | 0 h | 21.286175198 |
| NPB | *OsUBQ* | 0 h | 21.070917006 |
| NPB | *OsUBQ* | 2 h | 21.037236373 |
| NPB | *OsUBQ* | 2 h | 20.861614490 |
| NPB | *OsUBQ* | 2 h | 20.819745384 |
| NPB | *OsUBQ* | 6 h | 20.568748131 |
| NPB | *OsUBQ* | 6 h | 20.849152920 |
| NPB | *OsUBQ* | 6 h | 21.367222135 |
| NPB | *OsUBQ* | 12 h | 25.688672599 |
| NPB | *OsUBQ* | 12 h | 25.647627434 |
| NPB | *OsUBQ* | 12 h | 25.821776060 |
| NPB | *OsWHY1* | 0 h | 29.909614300 |
| NPB | *OsWHY1* | 0 h | 29.894024084 |
| NPB | *OsWHY1* | 0 h | 30.184093477 |
| NPB | *OsWHY1* | 2 h | 28.088426646 |
| NPB | *OsWHY1* | 2 h | 27.737887753 |
| NPB | *OsWHY1* | 2 h | 28.132165405 |
| NPB | *OsWHY1* | 6 h | 28.699530840 |
| NPB | *OsWHY1* | 6 h | 29.132631352 |
| NPB | *OsWHY1* | 6 h | 29.381632516 |
| NPB | *OsWHY1* | 12 h | 33.582027909 |
| NPB | *OsWHY1* | 12 h | 33.050698547 |
| NPB | *OsWHY1* | 12 h | 32.846700451 |

**SA treatment**

| **background** | **Abbreviation** | **Hours post-treatment** | **Raw Ct values** |
| --- | --- | --- | --- |
| NPB | *OsUBQ* | 0 h | 21.192348257 |
| NPB | *OsUBQ* | 0 h | 21.082652635 |
| NPB | *OsUBQ* | 0 h | 21.362126375 |
| NPB | *OsUBQ* | 2 h | 20.316618738 |
| NPB | *OsUBQ* | 2 h | 20.164199938 |
| NPB | *OsUBQ* | 2 h | 20.276746502 |
| NPB | *OsUBQ* | 6 h | 23.344106715 |
| NPB | *OsUBQ* | 6 h | 23.688929159 |
| NPB | *OsUBQ* | 6 h | 23.590766696 |
| NPB | *OsUBQ* | 12 h | 18.781140155 |
| NPB | *OsUBQ* | 12 h | 18.737369923 |
| NPB | *OsUBQ* | 12 h | 18.716895197 |
| NPB | *OsWHY1* | 0 h | 29.888213746 |
| NPB | *OsWHY1* | 0 h | 29.903041424 |
| NPB | *OsWHY1* | 0 h | 29.953434536 |
| NPB | *OsWHY1* | 2 h | 30.246338578 |
| NPB | *OsWHY1* | 2 h | 29.848808670 |
| NPB | *OsWHY1* | 2 h | 30.023551693 |
| NPB | *OsWHY1* | 6 h | 31.231642521 |
| NPB | *OsWHY1* | 6 h | 31.508627187 |
| NPB | *OsWHY1* | 6 h | 31.187379098 |
| NPB | *OsWHY1* | 12 h | 26.802596302 |
| NPB | *OsWHY1* | 12 h | 27.077092026 |
| NPB | *OsWHY1* | 12 h | 26.399098915 |

**Flg22 treatment**

| **background** | **Abbreviation** | **Hours post-treatment** | **Raw Ct values** |
| --- | --- | --- | --- |
| NPB | *OsUBQ* | 0 h | 22.577603994 |
| NPB | *OsUBQ* | 0 h | 22.520830299 |
| NPB | *OsUBQ* | 0 h | 22.772634354 |
| NPB | *OsUBQ* | 0.5 h | 24.678579144 |
| NPB | *OsUBQ* | 0.5 h | 24.681549354 |
| NPB | *OsUBQ* | 0.5 h | 24.798520274 |
| NPB | *OsUBQ* | 1 h | 26.224147843 |
| NPB | *OsUBQ* | 1 h | 26.006080012 |
| NPB | *OsUBQ* | 1 h | 25.913491192 |
| NPB | *OsUBQ* | 3 h | 25.592552841 |
| NPB | *OsUBQ* | 3 h | 25.613326202 |
| NPB | *OsUBQ* | 3 h | 28.778479977 |
| NPB | *OsWHY1* | 0 h | 29.559497438 |
| NPB | *OsWHY1* | 0 h | 29.068079499 |
| NPB | *OsWHY1* | 0 h | 29.974495042 |
| NPB | *OsWHY1* | 0.5 h | 30.76159892 |
| NPB | *OsWHY1* | 0.5 h | 30.621432045 |
| NPB | *OsWHY1* | 0.5 h | 30.635683911 |
| NPB | *OsWHY1* | 1 h | 32.268388019 |
| NPB | *OsWHY1* | 1 h | 32.458864776 |
| NPB | *OsWHY1* | 1 h | 32.887272462 |
| NPB | *OsWHY1* | 3 h | 31.125705356 |
| NPB | *OsWHY1* | 3 h | 31.19889952 |
| NPB | *OsWHY1* | 3 h | 31.244164876 |

**chitin treatment**

| **background** | **Abbreviation** | **Hours post-treatment** | **Raw Ct values** |
| --- | --- | --- | --- |
| NPB | *OsUBQ* | 0 h | 23.498179492 |
| NPB | *OsUBQ* | 0 h | 23.524654910 |
| NPB | *OsUBQ* | 0 h | 23.333735502 |
| NPB | *OsUBQ* | 0.5 h | 20.162660418 |
| NPB | *OsUBQ* | 0.5 h | 20.122534073 |
| NPB | *OsUBQ* | 0.5 h | 20.011133808 |
| NPB | *OsUBQ* | 1 h | 25.446945351 |
| NPB | *OsUBQ* | 1 h | 25.576403065 |
| NPB | *OsUBQ* | 1 h | 26.368822547 |
| NPB | *OsUBQ* | 3 h | 20.447821928 |
| NPB | *OsUBQ* | 3 h | 20.135953491 |
| NPB | *OsUBQ* | 3 h | 20.018754913 |
| NPB | *OsWHY1* | 0 h | 29.338010256 |
| NPB | *OsWHY1* | 0 h | 29.808600926 |
| NPB | *OsWHY1* | 0 h | 29.128287622 |
| NPB | *OsWHY1* | 0.5 h | 28.511885739 |
| NPB | *OsWHY1* | 0.5 h | 28.735309311 |
| NPB | *OsWHY1* | 0.5 h | 28.202950070 |
| NPB | *OsWHY1* | 1 h | 32.491522923 |
| NPB | *OsWHY1* | 1 h | 31.561529191 |
| NPB | *OsWHY1* | 1 h | 32.333754639 |
| NPB | *OsWHY1* | 3 h | 30.180116946 |
| NPB | *OsWHY1* | 3 h | 30.029106533 |
| NPB | *OsWHY1* | 3 h | 30.138979533 |

***Magnaporthe oryzae* inoculation**

| **background** | **Abbreviation** | **Hours post-inoculation** | **Raw Ct values** |
| --- | --- | --- | --- |
| NPB | *OsUBQ* | 0 h | 32.904123693 |
| NPB | *OsUBQ* | 0 h | 32.860052127 |
| NPB | *OsUBQ* | 0 h | 32.615411639 |
| NPB | *OsUBQ* | 24 h | 33.320163740 |
| NPB | *OsUBQ* | 24 h | 33.515794292 |
| NPB | *OsUBQ* | 24 h | 33.537890605 |
| NPB | *OsUBQ* | 48 h | 29.997738462 |
| NPB | *OsUBQ* | 48 h | 29.920003435 |
| NPB | *OsUBQ* | 48 h | 30.049801230 |
| NPB | *OsUBQ* | 72 h | 29.935849947 |
| NPB | *OsUBQ* | 72 h | 29.823198040 |
| NPB | *OsUBQ* | 72 h | 29.722994919 |
| NPB | *OsWHY1* | 0 h | 28.481420318 |
| NPB | *OsWHY1* | 0 h | 28.308916436 |
| NPB | *OsWHY1* | 0 h | 28.010960530 |
| NPB | *OsWHY1* | 24 h | 27.275672000 |
| NPB | *OsWHY1* | 24 h | 27.546026523 |
| NPB | *OsWHY1* | 24 h | 27.165669925 |
| NPB | *OsWHY1* | 48 h | 26.779009726 |
| NPB | *OsWHY1* | 48 h | 27.103049850 |
| NPB | *OsWHY1* | 48 h | 27.028770752 |
| NPB | *OsWHY1* | 72 h | 30.394498964 |
| NPB | *OsWHY1* | 72 h | 30.371229752 |
| NPB | *OsWHY1* | 72 h | 30.294317904 |

**Planta fungal growth**

| **background** | **Abbreviation** | **Day post-inoculation** | **Raw Ct values** |
| --- | --- | --- | --- |
| NPB | *OsUBQ* | 5 dpi | 22.028512782 |
| NPB | *OsUBQ* | 5 dpi | 22.222826117 |
| NPB | *OsUBQ* | 5 dpi | 21.977546699 |
| NPB | *MoPOT2* | 5 dpi | 28.31329729 |
| NPB | *MoPOT2* | 5 dpi | 28.16724297 |
| NPB | *MoPOT2* | 5 dpi | 28.262695795 |
| *OsWHY1*-OX-4 | *OsUBQ* | 5 dpi | 24.450962416 |
| *OsWHY1*-OX-4 | *OsUBQ* | 5 dpi | 24.624870161 |
| *OsWHY1*-OX-4 | *OsUBQ* | 5 dpi | 24.578999187 |
| *OsWHY1*-OX-4 | *MoPOT2* | 5 dpi | 28.021590223 |
| *OsWHY1*-OX-4 | *MoPOT2* | 5 dpi | 28.096957414 |
| *OsWHY1*-OX-4 | *MoPOT2* | 5 dpi | 28.144332156 |
| *OsWHY1*-OX-18 | *OsUBQ* | 5 dpi | 24.152832636 |
| *OsWHY1*-OX-18 | *OsUBQ* | 5 dpi | 24.262784392 |
| *OsWHY1*-OX-18 | *OsUBQ* | 5 dpi | 24.243536618 |
| *OsWHY1*-OX-18 | *MoPOT2* | 5 dpi | 28.052459542 |
| *OsWHY1*-OX-18 | *MoPOT2* | 5 dpi | 28.025269424 |
| *OsWHY1*-OX-18 | *MoPOT2* | 5 dpi | 28.002607767 |

***Magnaporthe oryzae* inoculation**

| **background** | **Abbreviation** | **Hours post-treatment** | **Raw Ct values** |
| --- | --- | --- | --- |
| NPB | *OsUBQ* | 0 h | 23.866582279 |
| NPB | *OsUBQ* | 0 h | 23.704757124 |
| NPB | *OsUBQ* | 0 h | 23.517910006 |
| NPB | *OsUBQ* | 12 h | 26.923384461 |
| NPB | *OsUBQ* | 12 h | 27.024245539 |
| NPB | *OsUBQ* | 12 h | 26.877646818 |
| NPB | *OsUBQ* | 24 h | 26.573098860 |
| NPB | *OsUBQ* | 24 h | 26.804347413 |
| NPB | *OsUBQ* | 24 h | 26.624747910 |
| NPB | *OsUBQ* | 48 h | 28.666354330 |
| NPB | *OsUBQ* | 48 h | 28.248067163 |
| NPB | *OsUBQ* | 48 h | 28.250036908 |
| NPB | *OsUBQ* | 72 h | 25.128413851 |
| NPB | *OsUBQ* | 72 h | 25.217432239 |
| NPB | *OsUBQ* | 72 h | 25.181849072 |
| NPB | *OsWAK1* | 0 h | 32.482852393 |
| NPB | *OsWAK1* | 0 h | 32.755904738 |
| NPB | *OsWAK1* | 0 h | 32.267529283 |
| NPB | *OsWAK1* | 12 h | 32.847313147 |
| NPB | *OsWAK1* | 12 h | 32.623346518 |
| NPB | *OsWAK1* | 12 h | 32.786492049 |
| NPB | *OsWAK1* | 24 h | 29.385035207 |
| NPB | *OsWAK1* | 24 h | 29.051354742 |
| NPB | *OsWAK1* | 24 h | 28.766293187 |
| NPB | *OsWAK1* | 48 h | 31.935067278 |
| NPB | *OsWAK1* | 48 h | 31.750963126 |
| NPB | *OsWAK1* | 48 h | 31.743151879 |
| NPB | *OsWAK1* | 72 h | 31.378868141 |
| NPB | *OsWAK1* | 72 h | 31.664563566 |
| NPB | *OsWAK1* | 72 h | 31.412759817 |
| *OsWHY1*-OX | *OsUBQ* | 0 h | 27.227695481 |
| *OsWHY1*-OX | *OsUBQ* | 0 h | 26.871622632 |
| *OsWHY1*-OX | *OsUBQ* | 0 h | 27.066888182 |
| *OsWHY1*-OX | *OsUBQ* | 12 h | 26.253794597 |
| *OsWHY1*-OX | *OsUBQ* | 12 h | 26.359364949 |
| *OsWHY1*-OX | *OsUBQ* | 12 h | 26.403702343 |
| *OsWHY1*-OX | *OsUBQ* | 24 h | 27.137245249 |
| *OsWHY1*-OX | *OsUBQ* | 24 h | 27.427357526 |
| *OsWHY1*-OX | *OsUBQ* | 24 h | 27.008589582 |
| *OsWHY1*-OX | *OsUBQ* | 48 h | 27.978569688 |
| *OsWHY1*-OX | *OsUBQ* | 48 h | 27.818341952 |
| *OsWHY1*-OX | *OsUBQ* | 48 h | 27.564009965 |
| *OsWHY1*-OX | *OsUBQ* | 72 h | 27.781887325 |
| *OsWHY1*-OX | *OsUBQ* | 72 h | 27.768596108 |
| *OsWHY1*-OX | *OsUBQ* | 72 h | 27.139315009 |
| *OsWHY1*-OX | *OsWAK1* | 0 h | 32.086426008 |
| *OsWHY1*-OX | *OsWAK1* | 0 h | 32.564011707 |
| *OsWHY1*-OX | *OsWAK1* | 0 h | 32.487645660 |
| *OsWHY1*-OX | *OsWAK1* | 12 h | 31.443643428 |
| *OsWHY1*-OX | *OsWAK1* | 12 h | 31.603988363 |
| *OsWHY1*-OX | *OsWAK1* | 12 h | 31.850577541 |
| *OsWHY1*-OX | *OsWAK1* | 24 h | 35.636012793 |
| *OsWHY1*-OX | *OsWAK1* | 24 h | 37.031707674 |
| *OsWHY1*-OX | *OsWAK1* | 24 h | 36.614853534 |
| *OsWHY1*-OX | *OsWAK1* | 48 h | 32.265646552 |
| *OsWHY1*-OX | *OsWAK1* | 48 h | 32.275808592 |
| *OsWHY1*-OX | *OsWAK1* | 48 h | 32.255881230 |
| *OsWHY1*-OX | *OsWAK1* | 72 h | 33.470486246 |
| *OsWHY1*-OX | *OsWAK1* | 72 h | 33.343105895 |
| *OsWHY1*-OX | *OsWAK1* | 72 h | 33.323123276 |

***Magnaporthe oryzae* inoculation**

| **background** | **Abbreviation** | **Hours post-treatment** | **Raw Ct values** |
| --- | --- | --- | --- |
| NPB | *OsUBQ* | 0 h | 27.472886783 |
| NPB | *OsUBQ* | 0 h | 27.270785867 |
| NPB | *OsUBQ* | 0 h | 26.885652676 |
| NPB | *OsUBQ* | 12 h | 28.804784130 |
| NPB | *OsUBQ* | 12 h | 28.974693010 |
| NPB | *OsUBQ* | 12 h | 29.075708110 |
| NPB | *OsUBQ* | 24 h | 27.706996667 |
| NPB | *OsUBQ* | 24 h | 27.399223979 |
| NPB | *OsUBQ* | 24 h | 27.808670390 |
| NPB | *OsUBQ* | 48 h | 29.094593171 |
| NPB | *OsUBQ* | 48 h | 28.914467616 |
| NPB | *OsUBQ* | 48 h | 29.621981074 |
| NPB | *OsUBQ* | 72 h | 28.370901759 |
| NPB | *OsUBQ* | 72 h | 28.041862229 |
| NPB | *OsUBQ* | 72 h | 28.360143997 |
| NPB | *OsWRKY53* | 0 h | 28.705754685 |
| NPB | *OsWRKY53* | 0 h | 28.230660659 |
| NPB | *OsWRKY53* | 0 h | 28.525793739 |
| NPB | *OsWRKY53* | 12 h | 29.795697567 |
| NPB | *OsWRKY53* | 12 h | 29.848846157 |
| NPB | *OsWRKY53* | 12 h | 29.809884324 |
| NPB | *OsWRKY53* | 24 h | 28.906988705 |
| NPB | *OsWRKY53* | 24 h | 28.762517241 |
| NPB | *OsWRKY53* | 24 h | 28.271674056 |
| NPB | *OsWRKY53* | 48 h | 32.593627575 |
| NPB | *OsWRKY53* | 48 h | 32.584535509 |
| NPB | *OsWRKY53* | 48 h | 33.012218561 |
| NPB | *OsWRKY53* | 72 h | 30.447809334 |
| NPB | *OsWRKY53* | 72 h | 30.975320083 |
| NPB | *OsWRKY53* | 72 h | 30.230397931 |
| *OsWHY1*-OX | *OsUBQ* | 0 h | 27.650981656 |
| *OsWHY1*-OX | *OsUBQ* | 0 h | 27.688573316 |
| *OsWHY1*-OX | *OsUBQ* | 0 h | 27.175814410 |
| *OsWHY1*-OX | *OsUBQ* | 12 h | 28.938239561 |
| *OsWHY1*-OX | *OsUBQ* | 12 h | 28.536543713 |
| *OsWHY1*-OX | *OsUBQ* | 12 h | 28.866749790 |
| *OsWHY1*-OX | *OsUBQ* | 24 h | 28.068667351 |
| *OsWHY1*-OX | *OsUBQ* | 24 h | 28.976812187 |
| *OsWHY1*-OX | *OsUBQ* | 24 h | 28.162922702 |
| *OsWHY1*-OX | *OsUBQ* | 48 h | 28.290525680 |
| *OsWHY1*-OX | *OsUBQ* | 48 h | 27.935731557 |
| *OsWHY1*-OX | *OsUBQ* | 48 h | 28.058773287 |
| *OsWHY1*-OX | *OsUBQ* | 72 h | 30.091129255 |
| *OsWHY1*-OX | *OsUBQ* | 72 h | 30.443238187 |
| *OsWHY1*-OX | *OsUBQ* | 72 h | 30.505062048 |
| *OsWHY1*-OX | *OsWRKY53* | 0 h | 29.439460724 |
| *OsWHY1*-OX | *OsWRKY53* | 0 h | 29.267629976 |
| *OsWHY1*-OX | *OsWRKY53* | 0 h | 28.982009348 |
| *OsWHY1*-OX | *OsWRKY53* | 12 h | 30.598084108 |
| *OsWHY1*-OX | *OsWRKY53* | 12 h | 30.683501866 |
| *OsWHY1*-OX | *OsWRKY53* | 12 h | 31.285359236 |
| *OsWHY1*-OX | *OsWRKY53* | 24 h | 32.650806047 |
| *OsWHY1*-OX | *OsWRKY53* | 24 h | 33.449043944 |
| *OsWHY1*-OX | *OsWRKY53* | 24 h | 33.057790249 |
| *OsWHY1*-OX | *OsWRKY53* | 48 h | 30.542883727 |
| *OsWHY1*-OX | *OsWRKY53* | 48 h | 30.528246223 |
| *OsWHY1*-OX | *OsWRKY53* | 48 h | 30.019592935 |
| *OsWHY1*-OX | *OsWRKY53* | 72 h | 33.277956180 |
| *OsWHY1*-OX | *OsWRKY53* | 72 h | 34.289601808 |
| *OsWHY1*-OX | *OsWRKY53* | 72 h | 32.164577935 |

***Magnaporthe oryzae* inoculation**

| **background** | **Abbreviation** | **Hours post-treatment** | **Raw Ct values** |
| --- | --- | --- | --- |
| NPB | *OsUBQ* | 0 h | 26.262614244 |
| NPB | *OsUBQ* | 0 h | 26.497417313 |
| NPB | *OsUBQ* | 0 h | 26.906252092 |
| NPB | *OsUBQ* | 12 h | 27.233889946 |
| NPB | *OsUBQ* | 12 h | 26.975911800 |
| NPB | *OsUBQ* | 12 h | 27.222982455 |
| NPB | *OsUBQ* | 24 h | 27.170666947 |
| NPB | *OsUBQ* | 24 h | 27.151947560 |
| NPB | *OsUBQ* | 24 h | 26.871463031 |
| NPB | *OsUBQ* | 48 h | 27.158807618 |
| NPB | *OsUBQ* | 48 h | 27.079302823 |
| NPB | *OsUBQ* | 48 h | 27.364364984 |
| NPB | *OsUBQ* | 72 h | 23.453628654 |
| NPB | *OsUBQ* | 72 h | 23.410965346 |
| NPB | *OsUBQ* | 72 h | 23.253500248 |
| NPB | *OsSAG12* | 0 h | 26.960095454 |
| NPB | *OsSAG12* | 0 h | 26.540142487 |
| NPB | *OsSAG12* | 0 h | 26.712439379 |
| NPB | *OsSAG12* | 12 h | 27.371929958 |
| NPB | *OsSAG12* | 12 h | 27.337116974 |
| NPB | *OsSAG12* | 12 h | 27.559230027 |
| NPB | *OsSAG12* | 24 h | 26.087309488 |
| NPB | *OsSAG12* | 24 h | 25.868107884 |
| NPB | *OsSAG12* | 24 h | 25.902752960 |
| NPB | *OsSAG12* | 48 h | 27.120401694 |
| NPB | *OsSAG12* | 48 h | 27.235089772 |
| NPB | *OsSAG12* | 48 h | 27.127915337 |
| NPB | *OsSAG12* | 72 h | 27.054341591 |
| NPB | *OsSAG12* | 72 h | 27.468896705 |
| NPB | *OsSAG12* | 72 h | 27.329715378 |
| *OsWHY1*-OX | *OsUBQ* | 0 h | 28.341563028 |
| *OsWHY1*-OX | *OsUBQ* | 0 h | 28.362724194 |
| *OsWHY1*-OX | *OsUBQ* | 0 h | 28.223698720 |
| *OsWHY1*-OX | *OsUBQ* | 12 h | 27.736026042 |
| *OsWHY1*-OX | *OsUBQ* | 12 h | 27.360457939 |
| *OsWHY1*-OX | *OsUBQ* | 12 h | 27.831570245 |
| *OsWHY1*-OX | *OsUBQ* | 24 h | 26.228447496 |
| *OsWHY1*-OX | *OsUBQ* | 24 h | 26.213917905 |
| *OsWHY1*-OX | *OsUBQ* | 24 h | 26.024465989 |
| *OsWHY1*-OX | *OsUBQ* | 48 h | 27.343884357 |
| *OsWHY1*-OX | *OsUBQ* | 48 h | 27.601992368 |
| *OsWHY1*-OX | *OsUBQ* | 48 h | 27.409190040 |
| *OsWHY1*-OX | *OsUBQ* | 72 h | 28.141675576 |
| *OsWHY1*-OX | *OsUBQ* | 72 h | 27.549033286 |
| *OsWHY1*-OX | *OsUBQ* | 72 h | 27.338020786 |
| *OsWHY1*-OX | *OsSAG12* | 0 h | 27.115572198 |
| *OsWHY1*-OX | *OsSAG12* | 0 h | 26.947421885 |
| *OsWHY1*-OX | *OsSAG12* | 0 h | 27.112010366 |
| *OsWHY1*-OX | *OsSAG12* | 12 h | 27.405508384 |
| *OsWHY1*-OX | *OsSAG12* | 12 h | 27.256951272 |
| *OsWHY1*-OX | *OsSAG12* | 12 h | 27.685803925 |
| *OsWHY1*-OX | *OsSAG12* | 24 h | 26.446643006 |
| *OsWHY1*-OX | *OsSAG12* | 24 h | 26.390354559 |
| *OsWHY1*-OX | *OsSAG12* | 24 h | 26.477163122 |
| *OsWHY1*-OX | *OsSAG12* | 48 h | 28.525914816 |
| *OsWHY1*-OX | *OsSAG12* | 48 h | 28.428353498 |
| *OsWHY1*-OX | *OsSAG12* | 48 h | 28.531175121 |
| *OsWHY1*-OX | *OsSAG12* | 72 h | 28.882675306 |
| *OsWHY1*-OX | *OsSAG12* | 72 h | 28.526602269 |
| *OsWHY1*-OX | *OsSAG12* | 72 h | 28.725886193 |

***Magnaporthe oryzae* inoculation**

| **background** | **Abbreviation** | **Hours post-treatment** | **Raw Ct values** |
| --- | --- | --- | --- |
| NPB | *OsUBQ* | 0 h | 25.260582659 |
| NPB | *OsUBQ* | 0 h | 25.985684828 |
| NPB | *OsUBQ* | 0 h | 25.835091615 |
| NPB | *OsUBQ* | 12 h | 25.952846710 |
| NPB | *OsUBQ* | 12 h | 26.539495569 |
| NPB | *OsUBQ* | 12 h | 26.457240931 |
| NPB | *OsUBQ* | 24 h | 26.065710268 |
| NPB | *OsUBQ* | 24 h | 25.909928544 |
| NPB | *OsUBQ* | 24 h | 29.041950799 |
| NPB | *OsUBQ* | 48 h | 26.590868527 |
| NPB | *OsUBQ* | 48 h | 26.826847477 |
| NPB | *OsUBQ* | 48 h | 27.062826426 |
| NPB | *OsUBQ* | 72 h | 22.615251508 |
| NPB | *OsUBQ* | 72 h | 23.187931681 |
| NPB | *OsUBQ* | 72 h | 23.494921830 |
| NPB | *OsHSP58.7* | 0 h | 30.966923095 |
| NPB | *OsHSP58.7* | 0 h | 31.060275193 |
| NPB | *OsHSP58.7* | 0 h | 31.440861428 |
| NPB | *OsHSP58.7* | 12 h | 31.499586171 |
| NPB | *OsHSP58.7* | 12 h | 31.534868718 |
| NPB | *OsHSP58.7* | 12 h | 31.464303624 |
| NPB | *OsHSP58.7* | 24 h | 27.709520699 |
| NPB | *OsHSP58.7* | 24 h | 28.215687787 |
| NPB | *OsHSP58.7* | 24 h | 28.065400692 |
| NPB | *OsHSP58.7* | 48 h | 30.587301116 |
| NPB | *OsHSP58.7* | 48 h | 30.166794925 |
| NPB | *OsHSP58.7* | 48 h | 29.882604985 |
| NPB | *OsHSP58.7* | 72 h | 28.865348510 |
| NPB | *OsHSP58.7* | 72 h | 29.393901310 |
| NPB | *OsHSP58.7* | 72 h | 28.709762504 |
| *OsWHY1*-OX | *OsUBQ* | 0 h | 27.362292067 |
| *OsWHY1*-OX | *OsUBQ* | 0 h | 27.665218287 |
| *OsWHY1*-OX | *OsUBQ* | 0 h | 27.150399803 |
| *OsWHY1*-OX | *OsUBQ* | 12 h | 27.273840846 |
| *OsWHY1*-OX | *OsUBQ* | 12 h | 27.023477601 |
| *OsWHY1*-OX | *OsUBQ* | 12 h | 26.784067322 |
| *OsWHY1*-OX | *OsUBQ* | 24 h | 24.879384979 |
| *OsWHY1*-OX | *OsUBQ* | 24 h | 24.898664297 |
| *OsWHY1*-OX | *OsUBQ* | 24 h | 25.251934520 |
| *OsWHY1*-OX | *OsUBQ* | 48 h | 43.277323871 |
| *OsWHY1*-OX | *OsUBQ* | 48 h | 34.464543535 |
| *OsWHY1*-OX | *OsUBQ* | 48 h | 33.852657539 |
| *OsWHY1*-OX | *OsUBQ* | 72 h | 26.701063879 |
| *OsWHY1*-OX | *OsUBQ* | 72 h | 26.701063879 |
| *OsWHY1*-OX | *OsUBQ* | 72 h | 26.701063879 |
| *OsWHY1*-OX | *OsHSP58.7* | 0 h | 30.607980737 |
| *OsWHY1*-OX | *OsHSP58.7* | 0 h | 31.197666530 |
| *OsWHY1*-OX | *OsHSP58.7* | 0 h | 30.023640596 |
| *OsWHY1*-OX | *OsHSP58.7* | 12 h | 29.711613509 |
| *OsWHY1*-OX | *OsHSP58.7* | 12 h | 29.975608815 |
| *OsWHY1*-OX | *OsHSP58.7* | 12 h | 29.748263561 |
| *OsWHY1*-OX | *OsHSP58.7* | 24 h | 29.262147743 |
| *OsWHY1*-OX | *OsHSP58.7* | 24 h | 28.672508321 |
| *OsWHY1*-OX | *OsHSP58.7* | 24 h | 28.510382996 |
| *OsWHY1*-OX | *OsHSP58.7* | 48 h | 37.212672302 |
| *OsWHY1*-OX | *OsHSP58.7* | 48 h | 38.111437133 |
| *OsWHY1*-OX | *OsHSP58.7* | 48 h | 37.662054717 |
| *OsWHY1*-OX | *OsHSP58.7* | 72 h | 30.720139698 |
| *OsWHY1*-OX | *OsHSP58.7* | 72 h | 31.147649891 |
| *OsWHY1*-OX | *OsHSP58.7* | 72 h | 31.575160085 |

***Magnaporthe oryzae* inoculation**

| **background** | **Abbreviation** | **Hours post-treatment** | **Raw Ct values** |
| --- | --- | --- | --- |
| NPB | *OsUBQ* | 0 h | 26.255219628 |
| NPB | *OsUBQ* | 0 h | 26.620605665 |
| NPB | *OsUBQ* | 0 h | 26.262537564 |
| NPB | *OsUBQ* | 12 h | 26.453834888 |
| NPB | *OsUBQ* | 12 h | 26.571101414 |
| NPB | *OsUBQ* | 12 h | 26.812435014 |
| NPB | *OsUBQ* | 24 h | 26.830226617 |
| NPB | *OsUBQ* | 24 h | 26.705426855 |
| NPB | *OsUBQ* | 24 h | 26.887225680 |
| NPB | *OsUBQ* | 48 h | 27.194005136 |
| NPB | *OsUBQ* | 48 h | 27.175813819 |
| NPB | *OsUBQ* | 48 h | 26.640295967 |
| NPB | *OsUBQ* | 72 h | 23.410270358 |
| NPB | *OsUBQ* | 72 h | 23.146899496 |
| NPB | *OsUBQ* | 72 h | 23.217942623 |
| NPB | *OsMYB1R* | 0 h | 35.164274299 |
| NPB | *OsMYB1R* | 0 h | 35.042670253 |
| NPB | *OsMYB1R* | 0 h | 34.382390188 |
| NPB | *OsMYB1R* | 12 h | 34.759761799 |
| NPB | *OsMYB1R* | 12 h | 33.209422437 |
| NPB | *OsMYB1R* | 12 h | 34.343054416 |
| NPB | *OsMYB1R* | 24 h | 34.587620563 |
| NPB | *OsMYB1R* | 24 h | 34.034823126 |
| NPB | *OsMYB1R* | 24 h | 34.311221845 |
| NPB | *OsMYB1R* | 48 h | 35.329688803 |
| NPB | *OsMYB1R* | 48 h | 35.274909719 |
| NPB | *OsMYB1R* | 48 h | 34.994522777 |
| NPB | *OsMYB1R* | 72 h | 33.701341548 |
| NPB | *OsMYB1R* | 72 h | 33.225147010 |
| NPB | *OsMYB1R* | 72 h | 32.490896872 |
| *OsWHY1*-OX | *OsUBQ* | 0 h | 28.169105062 |
| *OsWHY1*-OX | *OsUBQ* | 0 h | 27.837540483 |
| *OsWHY1*-OX | *OsUBQ* | 0 h | 28.658213346 |
| *OsWHY1*-OX | *OsUBQ* | 12 h | 27.024205689 |
| *OsWHY1*-OX | *OsUBQ* | 12 h | 27.173734211 |
| *OsWHY1*-OX | *OsUBQ* | 12 h | 27.383704312 |
| *OsWHY1*-OX | *OsUBQ* | 24 h | 25.355885538 |
| *OsWHY1*-OX | *OsUBQ* | 24 h | 25.270964723 |
| *OsWHY1*-OX | *OsUBQ* | 24 h | 25.191031865 |
| *OsWHY1*-OX | *OsUBQ* | 48 h | 27.313978135 |
| *OsWHY1*-OX | *OsUBQ* | 48 h | 26.798670785 |
| *OsWHY1*-OX | *OsUBQ* | 48 h | 26.958028623 |
| *OsWHY1*-OX | *OsUBQ* | 72 h | 27.325555761 |
| *OsWHY1*-OX | *OsUBQ* | 72 h | 26.868465256 |
| *OsWHY1*-OX | *OsUBQ* | 72 h | 27.071562442 |
| *OsWHY1*-OX | *OsMYB1R* | 0 h | 35.530348572 |
| *OsWHY1*-OX | *OsMYB1R* | 0 h | 34.597616579 |
| *OsWHY1*-OX | *OsMYB1R* | 0 h | 33.920011488 |
| *OsWHY1*-OX | *OsMYB1R* | 12 h | 34.054392661 |
| *OsWHY1*-OX | *OsMYB1R* | 12 h | 34.108708357 |
| *OsWHY1*-OX | *OsMYB1R* | 12 h | 34.000076966 |
| *OsWHY1*-OX | *OsMYB1R* | 24 h | 33.183153740 |
| *OsWHY1*-OX | *OsMYB1R* | 24 h | 33.390890843 |
| *OsWHY1*-OX | *OsMYB1R* | 24 h | 34.578970521 |
| *OsWHY1*-OX | *OsMYB1R* | 48 h | 34.707719946 |
| *OsWHY1*-OX | *OsMYB1R* | 48 h | 34.408712579 |
| *OsWHY1*-OX | *OsMYB1R* | 48 h | 35.762931253 |
| *OsWHY1*-OX | *OsMYB1R* | 72 h | 32.267840522 |
| *OsWHY1*-OX | *OsMYB1R* | 72 h | 33.115916157 |
| *OsWHY1*-OX | *OsMYB1R* | 72 h | 32.931819799 |

***Magnaporthe oryzae* inoculation**

| **background** | **Abbreviation** | **Hours post-treatment** | **Raw Ct values** |
| --- | --- | --- | --- |
| NPB | *OsUBQ* | 0 h | 24.714924901 |
| NPB | *OsUBQ* | 0 h | 24.692768540 |
| NPB | *OsUBQ* | 0 h | 24.533243900 |
| NPB | *OsUBQ* | 12 h | 29.048983041 |
| NPB | *OsUBQ* | 12 h | 28.981248458 |
| NPB | *OsUBQ* | 12 h | 29.724709980 |
| NPB | *OsUBQ* | 24 h | 29.015389981 |
| NPB | *OsUBQ* | 24 h | 29.176821365 |
| NPB | *OsUBQ* | 24 h | 29.267723521 |
| NPB | *OsUBQ* | 48 h | 29.481733184 |
| NPB | *OsUBQ* | 48 h | 30.697606235 |
| NPB | *OsUBQ* | 48 h | 30.398704726 |
| NPB | *OsUBQ* | 72 h | 26.225227434 |
| NPB | *OsUBQ* | 72 h | 25.942805122 |
| NPB | *OsUBQ* | 72 h | 26.410143972 |
| NPB | *OsPR2* | 0 h | 24.874754349 |
| NPB | *OsPR2* | 0 h | 24.726756095 |
| NPB | *OsPR2* | 0 h | 24.751464220 |
| NPB | *OsPR2* | 12 h | 26.176097428 |
| NPB | *OsPR2* | 12 h | 26.383871120 |
| NPB | *OsPR2* | 12 h | 26.207502707 |
| NPB | *OsPR2* | 24 h | 25.171133092 |
| NPB | *OsPR2* | 24 h | 25.192032459 |
| NPB | *OsPR2* | 24 h | 24.864400479 |
| NPB | *OsPR2* | 48 h | 27.668788115 |
| NPB | *OsPR2* | 48 h | 27.362202048 |
| NPB | *OsPR2* | 48 h | 27.518345751 |
| NPB | *OsPR2* | 72 h | 26.161595973 |
| NPB | *OsPR2* | 72 h | 26.161720011 |
| NPB | *OsPR2* | 72 h | 25.810488643 |
| *OsWHY1*-OX | *OsUBQ* | 0 h | 31.243648500 |
| *OsWHY1*-OX | *OsUBQ* | 0 h | 29.439691528 |
| *OsWHY1*-OX | *OsUBQ* | 0 h | 31.013169659 |
| *OsWHY1*-OX | *OsUBQ* | 12 h | 28.853567533 |
| *OsWHY1*-OX | *OsUBQ* | 12 h | 28.786711086 |
| *OsWHY1*-OX | *OsUBQ* | 12 h | 28.716745135 |
| *OsWHY1*-OX | *OsUBQ* | 24 h | 29.581367511 |
| *OsWHY1*-OX | *OsUBQ* | 24 h | 29.709527151 |
| *OsWHY1*-OX | *OsUBQ* | 24 h | 29.653618161 |
| *OsWHY1*-OX | *OsUBQ* | 48 h | 30.365670796 |
| *OsWHY1*-OX | *OsUBQ* | 48 h | 30.249597463 |
| *OsWHY1*-OX | *OsUBQ* | 48 h | 30.370325610 |
| *OsWHY1*-OX | *OsUBQ* | 72 h | 29.512607563 |
| *OsWHY1*-OX | *OsUBQ* | 72 h | 29.109362232 |
| *OsWHY1*-OX | *OsUBQ* | 72 h | 29.563820800 |
| *OsWHY1*-OX | *OsPR2* | 0 h | 26.720761961 |
| *OsWHY1*-OX | *OsPR2* | 0 h | 26.691129975 |
| *OsWHY1*-OX | *OsPR2* | 0 h | 26.931069723 |
| *OsWHY1*-OX | *OsPR2* | 12 h | 26.667975180 |
| *OsWHY1*-OX | *OsPR2* | 12 h | 26.685935984 |
| *OsWHY1*-OX | *OsPR2* | 12 h | 26.560067278 |
| *OsWHY1*-OX | *OsPR2* | 24 h | 27.038882607 |
| *OsWHY1*-OX | *OsPR2* | 24 h | 27.185620503 |
| *OsWHY1*-OX | *OsPR2* | 24 h | 26.874934635 |
| *OsWHY1*-OX | *OsPR2* | 48 h | 25.363330938 |
| *OsWHY1*-OX | *OsPR2* | 48 h | 25.353902906 |
| *OsWHY1*-OX | *OsPR2* | 48 h | 25.728732901 |
| *OsWHY1*-OX | *OsPR2* | 72 h | 27.337203750 |
| *OsWHY1*-OX | *OsPR2* | 72 h | 26.971653699 |
| *OsWHY1*-OX | *OsPR2* | 72 h | 27.206474691 |

***Magnaporthe oryzae* inoculation**

| **background** | **Abbreviation** | **Hours post-treatment** | **Raw Ct values** |
| --- | --- | --- | --- |
| NPB | *OsUBQ* | 0 h | 23.352797460 |
| NPB | *OsUBQ* | 0 h | 23.367287898 |
| NPB | *OsUBQ* | 0 h | 23.376681055 |
| NPB | *OsUBQ* | 12 h | 28.699729790 |
| NPB | *OsUBQ* | 12 h | 28.641736884 |
| NPB | *OsUBQ* | 12 h | 28.464621699 |
| NPB | *OsUBQ* | 24 h | 28.263577760 |
| NPB | *OsUBQ* | 24 h | 27.979014883 |
| NPB | *OsUBQ* | 24 h | 28.171040420 |
| NPB | *OsUBQ* | 48 h | 29.730018713 |
| NPB | *OsUBQ* | 48 h | 29.596082411 |
| NPB | *OsUBQ* | 48 h | 30.000489166 |
| NPB | *OsUBQ* | 72 h | 25.189821123 |
| NPB | *OsUBQ* | 72 h | 24.929333394 |
| NPB | *OsUBQ* | 72 h | 25.423412712 |
| NPB | *OsCATB* | 0 h | 26.042593454 |
| NPB | *OsCATB* | 0 h | 25.647849717 |
| NPB | *OsCATB* | 0 h | 26.457813527 |
| NPB | *OsCATB* | 12 h | 30.373382500 |
| NPB | *OsCATB* | 12 h | 30.056749468 |
| NPB | *OsCATB* | 12 h | 29.440431822 |
| NPB | *OsCATB* | 24 h | 27.276514247 |
| NPB | *OsCATB* | 24 h | 27.276835735 |
| NPB | *OsCATB* | 24 h | 27.501505429 |
| NPB | *OsCATB* | 48 h | 30.818002256 |
| NPB | *OsCATB* | 48 h | 30.197510143 |
| NPB | *OsCATB* | 48 h | 30.507350394 |
| NPB | *OsCATB* | 72 h | 26.729108039 |
| NPB | *OsCATB* | 72 h | 26.526842336 |
| NPB | *OsCATB* | 72 h | 26.200332566 |
| *OsWHY1*-OX | *OsUBQ* | 0 h | 30.014059829 |
| *OsWHY1*-OX | *OsUBQ* | 0 h | 29.224484989 |
| *OsWHY1*-OX | *OsUBQ* | 0 h | 29.176820215 |
| *OsWHY1*-OX | *OsUBQ* | 12 h | 29.675907341 |
| *OsWHY1*-OX | *OsUBQ* | 12 h | 29.285547415 |
| *OsWHY1*-OX | *OsUBQ* | 12 h | 29.725604745 |
| *OsWHY1*-OX | *OsUBQ* | 24 h | 26.802297711 |
| *OsWHY1*-OX | *OsUBQ* | 24 h | 27.784455209 |
| *OsWHY1*-OX | *OsUBQ* | 24 h | 28.135766680 |
| *OsWHY1*-OX | *OsUBQ* | 48 h | 27.740022918 |
| *OsWHY1*-OX | *OsUBQ* | 48 h | 28.120572649 |
| *OsWHY1*-OX | *OsUBQ* | 48 h | 27.656501395 |
| *OsWHY1*-OX | *OsUBQ* | 72 h | 29.045386269 |
| *OsWHY1*-OX | *OsUBQ* | 72 h | 29.998147400 |
| *OsWHY1*-OX | *OsUBQ* | 72 h | 29.337503274 |
| *OsWHY1*-OX | *OsCATB* | 0 h | 29.030178189 |
| *OsWHY1*-OX | *OsCATB* | 0 h | 28.899723865 |
| *OsWHY1*-OX | *OsCATB* | 0 h | 28.987214040 |
| *OsWHY1*-OX | *OsCATB* | 12 h | 30.200127246 |
| *OsWHY1*-OX | *OsCATB* | 12 h | 30.432254467 |
| *OsWHY1*-OX | *OsCATB* | 12 h | 30.027940550 |
| *OsWHY1*-OX | *OsCATB* | 24 h | 28.229850328 |
| *OsWHY1*-OX | *OsCATB* | 24 h | 28.227575265 |
| *OsWHY1*-OX | *OsCATB* | 24 h | 28.373137150 |
| *OsWHY1*-OX | *OsCATB* | 48 h | 27.696008095 |
| *OsWHY1*-OX | *OsCATB* | 48 h | 27.817758779 |
| *OsWHY1*-OX | *OsCATB* | 48 h | 28.146176702 |
| *OsWHY1*-OX | *OsCATB* | 72 h | 27.651998432 |
| *OsWHY1*-OX | *OsCATB* | 72 h | 27.641683353 |
| *OsWHY1*-OX | *OsCATB* | 72 h | 27.322677988 |

**Identification of overexpression lines**

| **background** | **Abbreviation** | **Raw Ct values** |
| --- | --- | --- |
| NPB | *OsUBQ* | 23.813060645 |
| NPB | *OsUBQ* | 23.862005401 |
| NPB | *OsUBQ* | 23.946453629 |
| NPB | *OsWHY1* | 30.590563871 |
| NPB | *OsWHY1* | 30.560216937 |
| NPB | *OsWHY1* | 30.580434773 |
| *OsWHY1*-OX-4 | *OsUBQ* | 25.305523634 |
| *OsWHY1*-OX-4 | *OsUBQ* | 25.293141332 |
| *OsWHY1*-OX-4 | *OsUBQ* | 25.465899305 |
| *OsWHY1*-OX-4 | *OsWHY1* | 25.051805120 |
| *OsWHY1*-OX-4 | *OsWHY1* | 25.226221749 |
| *OsWHY1*-OX-4 | *OsWHY1* | 25.316924960 |
| *OsWHY1*-OX-18 | *OsUBQ* | 25.629208396 |
| *OsWHY1*-OX-18 | *OsUBQ* | 25.950201143 |
| *OsWHY1*-OX-18 | *OsUBQ* | 26.141641503 |
| *OsWHY1*-OX-18 | *OsWHY1* | 24.227808660 |
| *OsWHY1*-OX-18 | *OsWHY1* | 24.185212476 |
| *OsWHY1*-OX-18 | *OsWHY1* | 24.570242216 |
